# SARS-CoV-2 infection following booster vaccination: illness and symptom profile in a prospective, observational community-based case-control study

**DOI:** 10.1016/j.jinf.2023.08.009

**Published:** 2023-09-28

**Authors:** Michela Antonelli, Rose S Penfold, Liane Dos Santos Canas, Carole Sudre, Khaled Rjoob, Ben Murray, Erika Molteni, Eric Kerfoot, Nathan Cheetham, Juan Capdevila Pujol, Lorenzo Polidori, Anna May, Jonathan Wolf, Marc Modat, Tim Spector, Alexander Hammers, Sebastien Ourselin, Claire Steves

**Affiliations:** 1 School of Biomedical Engineering & Imaging Sciences, King's College London, London, UK; 2 Ageing and Health Research Group, Usher Institute, University of Edinburgh, Edinburgh, UK; 3 Department of Twin Research and Genetic Epidemiology, King’s College London, UK; 4 Department of Ageing and Health, Guys and St Thomas’ NHS Foundation Trust, London, UK; 5 MRC Unit for Lifelong Health and Ageing at UCL, University College London, London, UK; 6 Centre for Medical Image Computing, University College London, London, UK; 7 Zoe Global, London, UK; 8 King's College London & Guy's and St Thomas' PET Centre

**Keywords:** COVID-19, vaccination, Omicron, infection, hospitalization, break-through infection, Long COVID, booster vaccination, disease severity

## Abstract

**Background:**

Booster COVID-19 vaccines have shown efficacy in clinical trials and effectiveness in real-world data against symptomatic and severe illness. However, some people still become infected with SARS-CoV-2 following a third (booster) vaccination. This study describes the characteristics of SARS-CoV-2 illness following a third vaccination and assesses the risk of progression to symptomatic disease in SARS-CoV-2 infected individuals with time since vaccination.

**Methods:**

This prospective, community-based, case-control study used data from UK-based, adult (≥18 years) users of the COVID Symptom Study mobile application, self-reporting a first positive COVID-19 test between June 1, 2021 and Apr 1, 2022. To describe the characteristics of SARS-CoV-2 illness following a third vaccination, we selected cases and controls who had received a third and second dose of monovalent vaccination against COVID-19 respectively and reported a first positive SARS-CoV-2 test at least 7 days after most recent vaccination. Cases and controls were matched (1:1) based on age, sex, BMI, time between first vaccination and infection, and week of testing. We used logistic regression models (adjusted for age, sex, BMI, level of social deprivation and frailty) to analyse associations of disease severity, overall disease duration, and individual symptoms with booster vaccination status. To assess potential waning of vaccine effectiveness, we compared disease severity, duration, and symptom profiles of individuals testing positive within 3 months of most recent vaccination (reference group) to profiles of individuals infected between 3-4, 4-5, and 5-6 months, for both third and second dose. All analyses were stratified by time period, based on the predominant SARS-CoV-2 variant at time of infection (Delta: June 1, 2021- 27 Nov, 2021; Omicron: 20 Dec, 2021-Apr 1, 2022).

**Findings:**

During the study period, 50,162 (Delta period) and 162,041 (Omicron) participants reported a positive SARS-CoV-2 test. During the Delta period, infection following three vaccination doses was associated with lower odds of long COVID (symptoms≥4weeks) (OR=0.83, CI[0.50-1.36], p<0.0001), hospitalization (OR=0.55, CI[0.39-0.75], p<0.0001) and severe symptoms (OR=0.36, CI[0.27-0.49], p<0.0001), and higher odds of asymptomatic infection (OR=3.45, CI[2.86-4.16], p<0.0001), compared to infection following only two vaccination doses. During the Omicron period, infection following three vaccination doses was associated with lower odds of severe symptoms (OR=0.48, CI[0.42-0.55], p<0.0001). During the Delta period, infected individuals were less likely to report almost all individual symptoms after a third vaccination. During the Omicron period, individuals were less likely to report most symptoms after a third vaccination, except for upper respiratory symptoms *e.g*. sneezing (OR=1.40, CI[1.18-1.35], p<0.0001), runny nose (OR=1.26, CI[1.18-1.35], p<0.0001), sore throat (OR=1.17, CI[1.10-1.25], p<0.0001), and hoarse voice (OR=1.13, CI[1.06-1.21], p<0.0001), which were more likely to be reported. There was evidence of reduced vaccine effectiveness during both Delta and Omicron periods in those infected more than 3 months after their most recent vaccination, with increased reporting of severe symptoms, long duration illness, and most individual symptoms.

**Interpretation:**

This study suggests that a third dose of monovalent vaccine may reduce symptoms, severity and duration of SARS-CoV-2 infection post-vaccination. For Omicron variants, third vaccination appears to reduce overall symptom burden, but may increase upper respiratory symptoms, potentially due to immunological priming. There is evidence of waning vaccine effectiveness against progression to symptomatic and severe disease and long COVID after three months. Our findings support ongoing booster vaccination promotion amongst individuals at high risk from COVID-19, to reduce severe symptoms and duration of illness, and health system burden. Disseminating knowledge on expected symptoms following booster vaccination may encourage vaccine uptake.

**Funding:**

This work is supported by UK Department of Health via the National Institute for Health Research (NIHR) comprehensive Biomedical Research Centre (BRC) award to Guy’s & St Thomas’ NHS Foundation Trust in partnership with King’s College London and King’s College Hospital NHS Foundation Trust and via a grant to ZOE Global; the Wellcome Engineering and Physical Sciences Research Council (EPSRC) Centre for Medical Engineering at King’s College London (WT 203148/Z/16/Z). Investigators also received support from the Chronic Disease Research Foundation, the Medical Research Council (MRC) [MR/R016372/1], British Heart Foundation, the National Institute for Health and Care Research (NIHR) [COV-LT-0009], the UK Research and Innovation London Medical Imaging & Artificial Intelligence Centre for Value Based Healthcare, the Wellcome Flagship Programme (WT213038/Z/18/Z and Alzheimer’s Society (AS-JF-17-011), and the Massachusetts Consortium on Pathogen Readiness (MassCPR). RSP is a fellow on the Multimorbidity Doctoral Training Programme for Health Professionals, which is supported by the Wellcome Trust [223499/Z/21/Z].

## Introduction

Booster vaccination remains a key strategy in response to the ongoing challenges posed by COVID-19. National UK government guidance at time of writing suggests that a seasonal booster should be offered to certain persons, including older adults and those in defined clinical risk groups.^1^ The aim of the national vaccination programme remains to reduce severe disease including hospitalization and mortality and prevent overburden of the National Health Service.

Several efficacy studies have demonstrated increased immunogenicity against SARS-CoV-2, including Omicron variants, following a booster vaccine.^2-6^ These *in vitro* findings are supported by real-world effectiveness studies, which have shown a lower risk of both asymptomatic and symptomatic infection with Omicron variants following booster vaccination.^7, 8^ Lower infection rates may translate to lower healthcare utilization, and a study across nine US states demonstrated that bivalent vaccines administered after at least 2 monovalent vaccine doses reduced COVID-19 related emergency attendances during the Omicron period.^9^

Nonetheless, some people still become infected with SARS-CoV-2 following three or more vaccine doses. A study of over one million older adults from US Veteran Health Administration facilities conducted across Delta and Omicron-predominant periods reported an incidence of breakthrough COVID-19 of 125.0 per 10, 000 persons following a booster vaccine, although notably the incidence of hospitalization or death was reported as low (8.9 per 10, 000 persons).^10^ To our knowledge, no studies have analysed the symptom profile of individuals infected with SARS-CoV-2 following three or more vaccine doses and there is little evidence on whether a third vaccine dose reduces the risk of prolonged symptoms (“long COVID”). In a previous study using data from over one million COVID Symptom Study participants, we described the characteristics of illness following the primary COVID-19 vaccination course, compared to illness in unvaccinated individuals.^11^ Vaccination was associated with a reduction in severe illness, and reduction in long-duration symptoms following the second vaccine dose. Almost all individual symptoms were less frequently reported in individuals infected post-vaccination, who were also more likely to be asymptomatic. The exception was sneezing, which was more common in infected individuals after the first vaccine dose. It remains unclear whether a third vaccine confers further protective effects against severe, symptomatic, and long duration disease.

Furthermore, accumulating evidence suggests a waning of vaccine effectiveness against symptomatic and severe illness with time since vaccination. Real-world population studies have suggested that efficacy of the primary vaccination course against severe illness (including hospitalization and death) may wane from around six months following second vaccination.^12, 13^ There is evidence that a booster may restore the protective effects of vaccination, with reductions in rates of infection, severe disease and hospitalization which persist for several months following booster vaccination.^14-18^ In a retrospective case control study of >30,000 participants aged over 65 years, Patalon *et al*. observed a significant waning in the relative protection of the BNT162b2 booster vaccine against the Omicron variant, from 53.4% one month after vaccination to 16.5% three months after vaccination.^19^ The authors suggested that a further booster dose may be needed to restore immunity in older adults. However, little is known about whether there is a waning of vaccine effectiveness against long duration illness (long COVID). This is important both for individual prognostication and to forecast longer-term healthcare utilization.

Elucidating disease severity, duration and symptom profiles in individuals infected with SARS-CoV-2 after booster vaccination has clinical importance, facilitating identification of groups to target for ongoing vaccination promotion efforts and intervention, and to forecast medical resource requirements.

This study aimed to:

1. Evaluate markers of illness severity and duration and assess symptom profile in individuals reporting SARS-CoV-2 infection after their third (“booster”) vaccination dose, compared to those reporting infection following two vaccine doses;
2. Investigate any change in the effectiveness of vaccination in reducing severity, symptoms and duration of illness related to SARS-CoV-2 infection over time following both booster and second vaccine doses.

## Methods

### Study design and participants

This prospective, community-based, case-control study used data from UK-based, adult (≥18 years) participants of the COVID Symptom Study logged through a free smartphone app developed by Zoe (London, UK) and King’s College London (London, UK). The app was launched in the UK on March 24, 2020.^20^ At registration, each participant reported baseline demographic information (e.g., age, sex, ethnicity, whether healthcare worker), geographic location, and information on health risk factors including comorbidities, lifestyle, frailty, and visits to hospital. Participants were encouraged to self-report any pre-specified symptoms daily, providing prospective, longitudinal information on incident symptoms. All users were prompted to record any COVID-19 testing results (whether tests were provided via the app or from other sources), and any COVID-19 vaccine(s) and subsequent symptoms.

Inclusion criteria for the study population were: 1) age ≥18 years; 2) living in the UK; 3) second or third vaccine dose received after June 1, 2021; 4) a positive reverse transcription polymerase chain reaction (RT-PCR) or lateral flow antigen test (LFAT) reported at least seven days after vaccination (in the case of multiple positive test results, only the first was selected). We excluded frontline healthcare workers (3.2% 7,493/234,951 users) due to differences in vaccination schedule and propensity to test as a result of occupational exposure to SARS-CoV-2.

We considered only positive tests reported after June 1, 2021, when the Delta variant was predominant in the UK (prevalence >70%) and up to April 1, 2022, when the UK government stopped providing coronavirus tests free-of-charge to the general public. Each included participant was at least seven days post-vaccination and had at least 12 weeks of symptom reporting following the positive test. Data census for symptom reporting was June 24, 2022 to allow each user at least 12 weeks of reporting.

To analyse the effect of booster vaccination on post-vaccination infection we compared disease severity, duration, and individual symptoms in users reporting a positive SARS-CoV-2 test at least seven days after a third vaccine dose (cases) to those reporting a positive test at least seven days after a second dose (controls). Controls were matched 1:1 with cases by a Euclidean-based algorithm based on age, sex, Body Mass Index (BMI), time elapsed between the first vaccination and the infection, and test week, a method used in our previously published study.^11, 21^

The following were assessed as indicators of disease severity: (i) self-reported hospitalization; (ii) reported acute, functionally limiting (“severe”) symptoms (two or more of fever, shortness of breath, and severe fatigue), and (iii) asymptomatic infection. For assessment of disease duration, we analysed associations of booster vaccination with reported symptoms lasting both ≥4 weeks, and ≥12 weeks. In analyses of symptom duration, we considered only users who logged using the app at least once per week for 4 weeks or longer after reporting a positive COVID-19 test.

For symptom profile analysis, symptoms reported between 3 days before and up to 14 days after the positive test date were considered, reflecting the acute phase of the disease. This window was used because it might have taken up to 3 days to request a RT-PCR test and receive a result following symptom onset, and symptoms can occur up to 14 days following SARS-CoV-2 exposure.^11, 22^ The full list of symptoms that can be reported using the app is shown in Supplementary Table 1. Symptoms were only included if they had a reported prevalence of more than 5% in the study population.

To assess for any waning of VE, we considered as a reference group participants infected within 3 months, and compared their disease severity, duration, and symptom profiles with participants infected between 3 and 4, 4 and 5, and 5 and 6 months following vaccination, for both third and second vaccine doses. We chose as the reference group individuals with Time Since Vaccination (TSV) <3 months as antibody levels have been demonstrated to significantly reduce by 3 months after 3rd vaccination.^23, 24^ Cases and controls were matched through a Euclidean-based algorithm based on age, sex, BMI, and test week.^11, 21^

We stratified all analyses into two time periods according to when Delta and Omicron variants of COVID-19 were predominant (>70%): 1st June, 2021-27th November, 2021 (Delta) and 20th December, 2021- 1st April, 2022 (Omicron).

### Statistical analyses

Data were extracted and preprocessed using ExeTera13, a Python library developed at KCL, and openly available on GitHub.^25^ Statistical analyses used Python 3.7 and the packages numpy v1.19.2, pandas v1.1.3, scipy 1.5.2, and statsmodels v0.12.1.

#### Disease severity, duration and symptom profile following booster versus second vaccine dose

To assess the effect of booster vaccination on disease severity, duration and symptom profile, we used univariate logistic regression models adjusted for age, sex, BMI, Index of Multiple Deprivation (IMD), and frailty. IMD is an area-based measure of relative deprivation. Frailty was assessed by the PRISMA7 questionnaire embedded in the app and classified as a binary variable (PRISMA7≥3 = frail; PRISMA7<3 = not frail).^26, 27^ For disease severity, we considered as outcomes the odds of self-reported: 1) hospitalization; 2) reporting acute functionally limiting symptoms, defined as two or more of fever, shortness of breath, and fatigue during the first 14 days, and 3) reporting no symptoms (asymptomatic). For duration, we considered as outcomes the presence of symptoms reported for ≥4 weeks and ≥12 weeks (post-COVID-19 syndrome). For symptom profile analysis, the outcome was the presence of each individual symptom.

#### Waning of vaccine effect

To assess for potential waning of VE against symptomatic or severe infection, we used the univariate logistic regression models as described above, comparing disease severity, duration and symptom profiles of individuals testing positive within 3 months of most recent vaccination (reference group) to profiles of individuals infected between 3-4, 4-5, and 5-6 months.

### Ethical approval

All app users provided informed consent for data usage for COVID-19-related research. In the UK, the app and study were approved by King’s College London’s (KCL) ethics committee (REMAS no. 18210, reference LRS-19/20–18210).

### Role of the funding source

Funders had no role in design, analysis, or interpretation of the data. Zoe Global, funded in part by the Department of Health and Social Care, made the app available for data collection as a not-for-profit endeavour.

## Results

During the Delta and Omicron periods, 50,162 and 162,041 users reported a first positive test for SARS-CoV-2, respectively. Of these, 1,910 (Delta period) and 154,057 (Omicron period) participants reported a positive test at least 7 days after the third vaccination dose, and 48,252 (Delta period) and 7,984 (Omicron period) reported a first positive test at least 7 days after the second vaccination. These differences are explained by the timing of Omicron relative to the booster vaccination program. Table 1 shows the demographic characteristics of cases and controls after 1:1 matching. During both periods, the majority of users were female (around 60%) and lived in areas with a greater level of social deprivation. For the Omicron period, controls were significantly younger (p<0·0001), and there was a significant difference in the number of people with frailty (higher for controls). There was no significant difference in the prevalence of comorbidities between the two groups.

### Disease severity, duration and symptom profile following booster versus second vaccine dose

**Figure 1:**
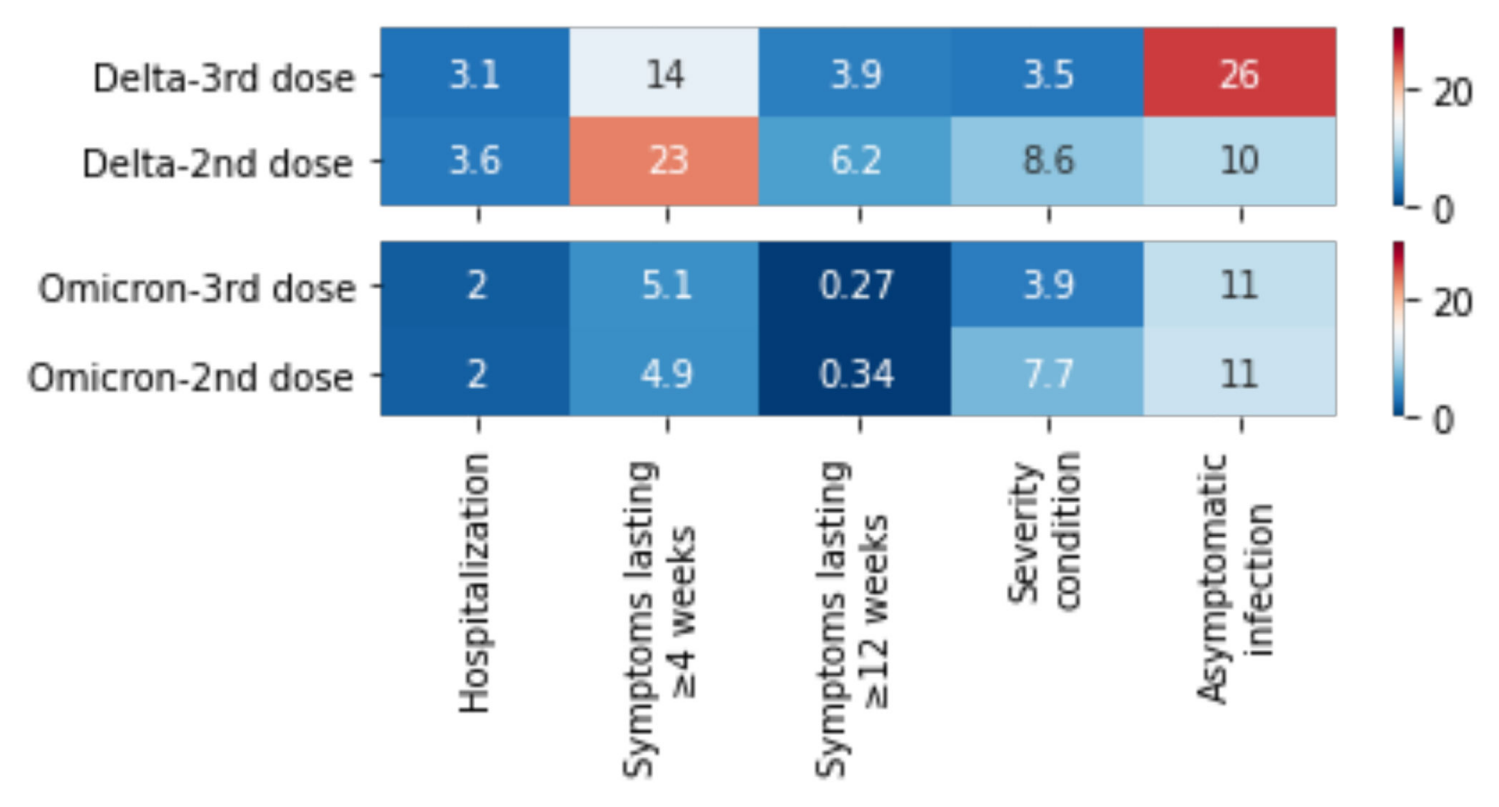
Proportion (%) of positive individuals in each time period with each severity outcome, stratified by dose and predominant variant

Before matching, during both Delta and Omicron periods, there was a lower proportion of individuals hospitalized, reporting severe symptoms (two or more of shortness of breath, fever and severe fatigue), and with duration of symptoms ≥ 12 weeks amongst individuals infected after 3 doses versus after 2 doses. During the Delta period, there was a substantially higher proportion of asymptomatic infections and a lower proportion of individuals with long COVID amongst cases versus controls. During the Omicron period, the proportion of individuals with asymptomatic infections and long COVID was similar amongst cases and controls, although a significantly higher proportion reported severe symptoms (Figure 1).

**Figure 2:**
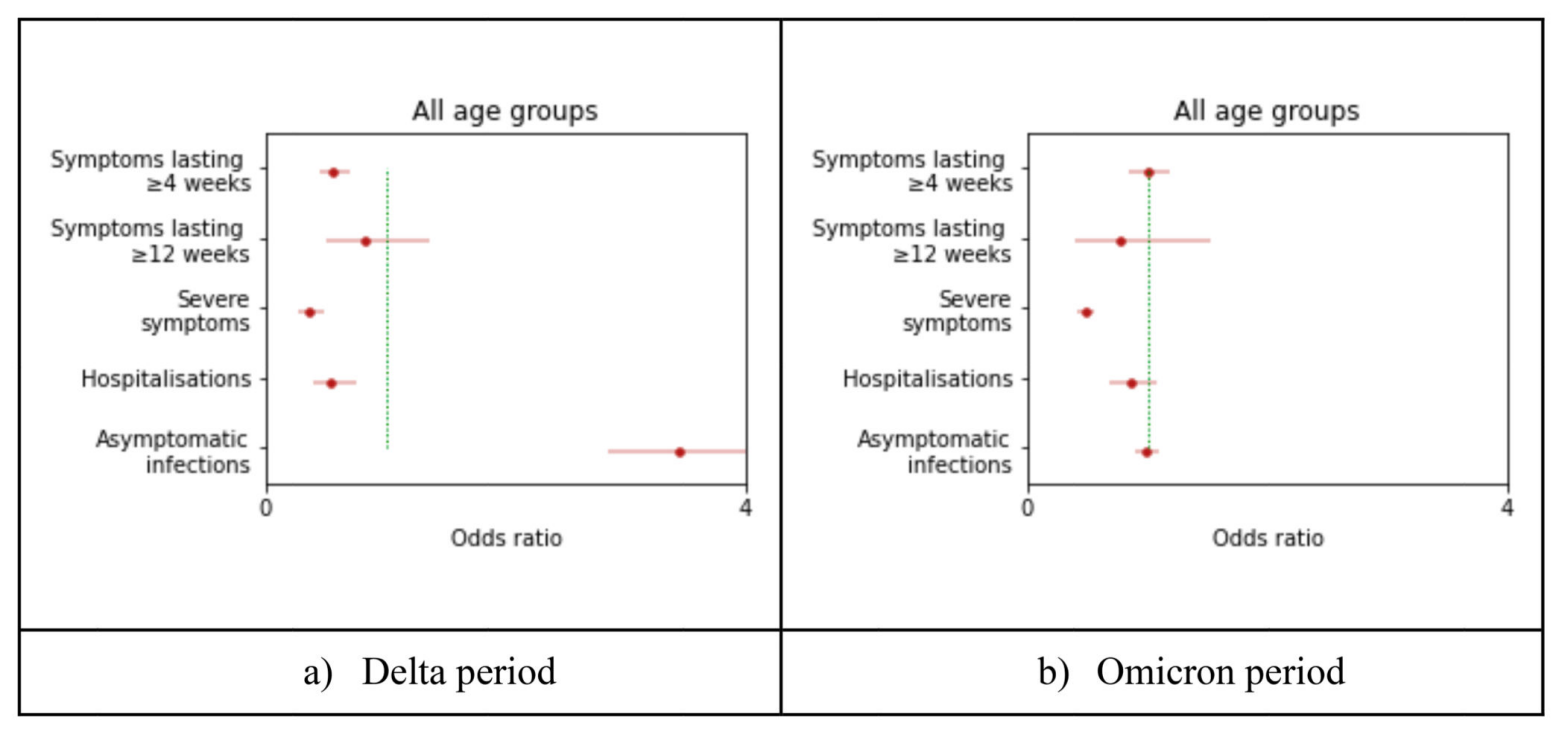
Odds ratio of asymptomatic infection, duration of symptoms ≥ 4 weeks, duration of symptoms ≥ 12 weeks, severe condition (two out of three among severe shortness of breath, fatigue, and fever), and hospitalization in app participants following booster vaccination vs 2nd dose, adjusted by age, BMI, sex, frailty, and IMD, stratified by variant.

In univariate matched case-control analyses adjusted for age, BMI, sex, frailty, and IMD, during the Delta period, there was a lower likelihood of self-reported hospitalization (OR=0.55, CI[0.39-0.75], p-value<0.0001), severe symptoms (OR=0.36, CI[0.27-0.49], p-value<0.0001) and long illness duration (≥4 weeks) (OR=0.56, CI[0.44-0.70], p-value<0.0001), and a higher likelihood of asymptomatic infection (OR=3.45, CI[2.86-4.16], p-value<0.0001) in cases versus controls (Figure 2a, Supplementary Table 2). During the Omicron period, there were lower odds of severe symptoms, (OR=0.48 CI[0.42-0.55], p-value<0.0001), but no difference in other indicators of disease severity in infection following a third vaccine dose (Figure 2b, Supplementary Table 3). The same results were obtained when the analysis was stratified by age group (Supplementary Figure 1).

**Figure 3:**
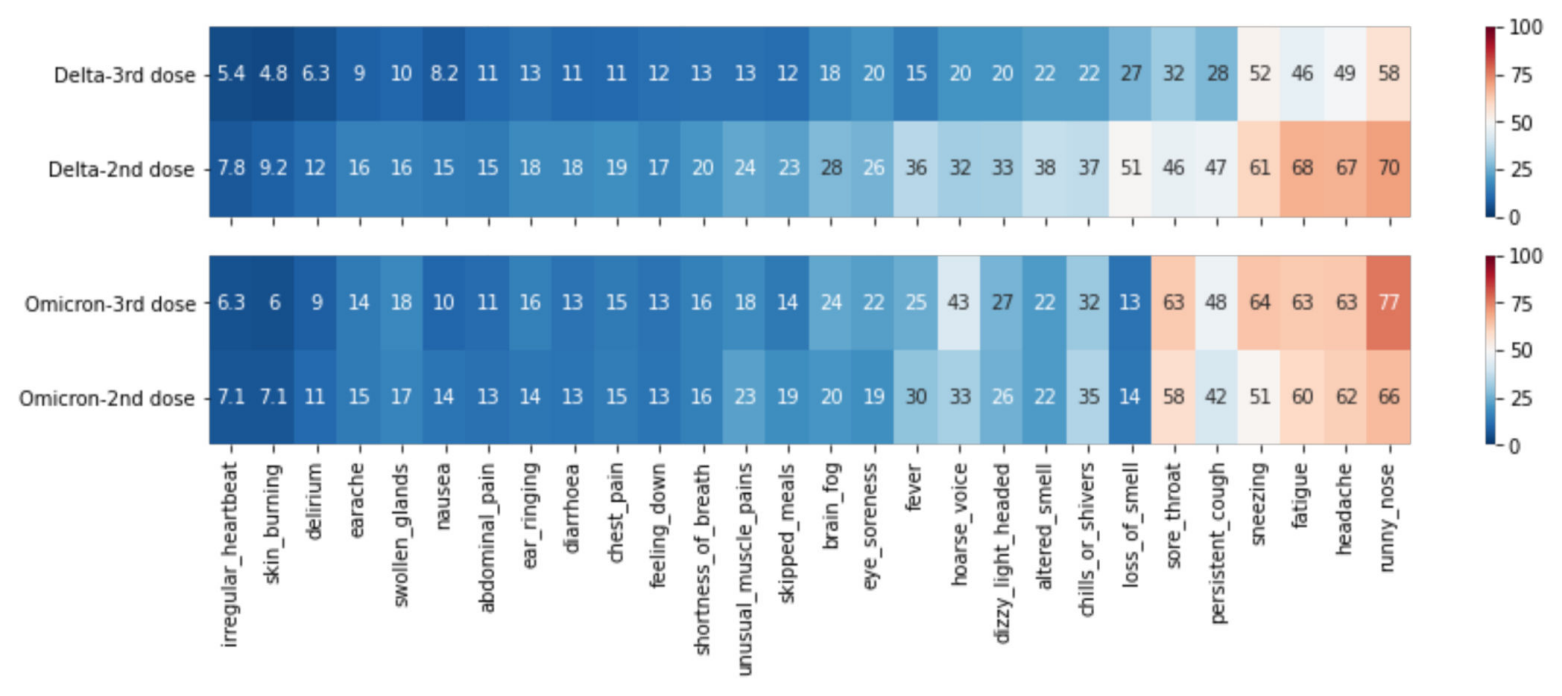
Heat map of proportion of infected participants (syntomatic and asymptomatic) reporting each symptom following 2nd and 3rd vaccination doses, stratified by variant.

During both periods, the most prevalent symptoms (reported by more than 50% of infected participants) were runny nose, headache, fatigue, and sneezing. During the Delta period, the proportion of infected individuals reporting almost all individual symptoms following a third vaccine was lower than following the second dose. During the Omicron period, the proportion of individuals reporting most symptoms was lower following a third vaccine, except for runny nose, sneezing, sore throat, and hoarse voice, which were more frequently reported (Figure 3).

**Figure 4:**
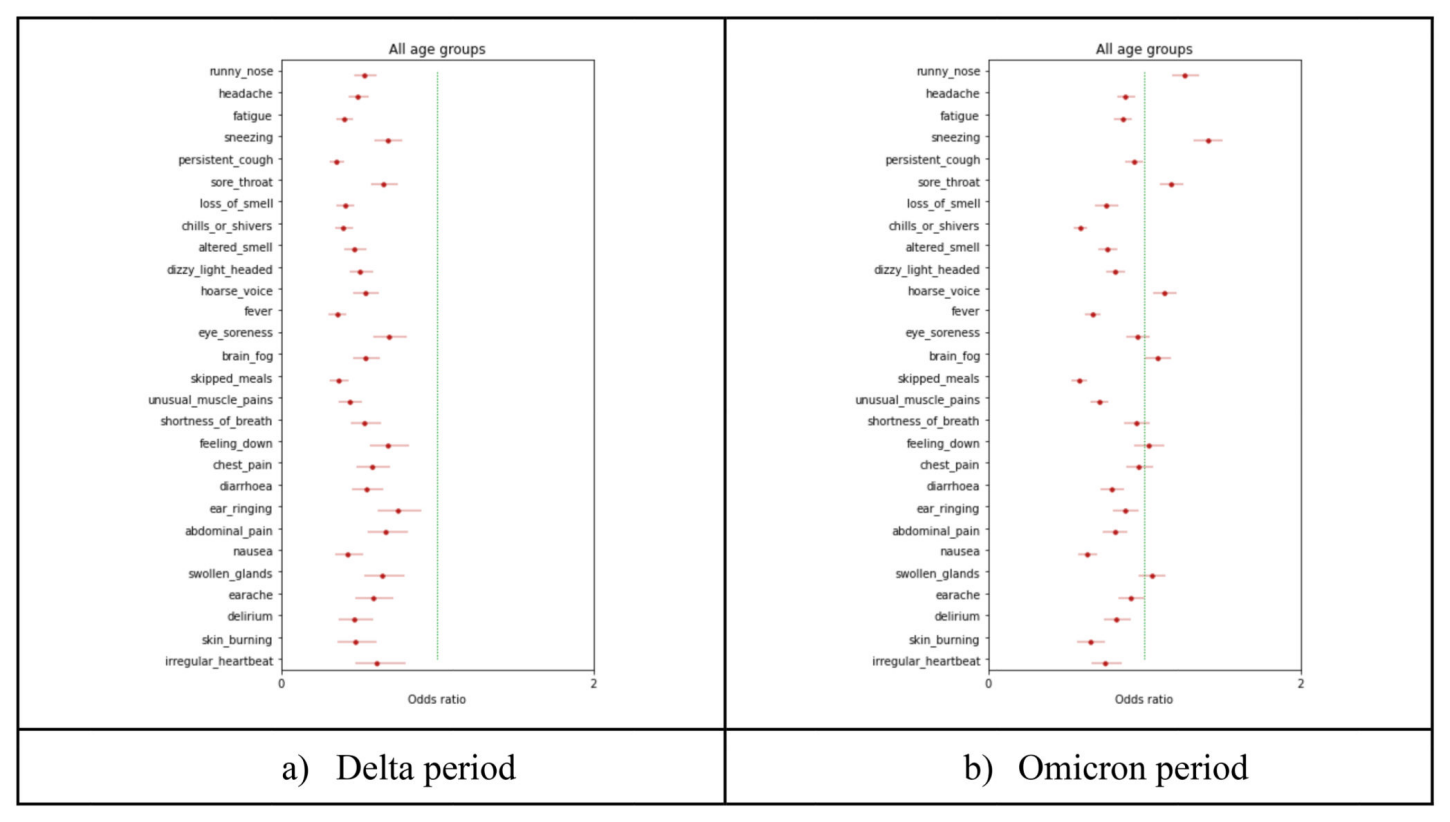
Odds Ratio of individual symptoms in individuals vaccinated with third dose versus individuals vaccinated with the second dose adjusted by age, BMI, sex, frailty, and IMD for the (a) Delta period and (b) Omicron period.

In the univariate matched case-control analysis of symptoms, adjusted for age, BMI, sex, frailty, and IMD, during the Delta period there was a significantly lower likelihood of all individual self-reported symptoms, with ORs ranging from 0.36 and 0.75 (Figure 4a and Supplementary Table 4). In contrast, during the Omicron period, only 18 out of 28 symptoms were significantly less frequently self-reported in individuals vaccinated with 3rd dose. Upper respiratory symptoms were significantly more likely to be self-reported in individuals vaccinated with 3rd dose (runny nose: OR=1.26, CI[1.18-1.69], p<0.0001; sore throat: OR=1.17 CI[1.10-1.25, p<0.0001; sneezing OR=1.40, CI[1.32-1.50], p<0.0001; hoarse voice: OR=1.13, CI=[1.06-1.21], p<0.000) (Figure 4b and Supplementary Table 5). Same results are obtained when the analysis is stratified by age groups (see Supplementary Figure 2).

### Waning of vaccine effect

**Figure 5:**
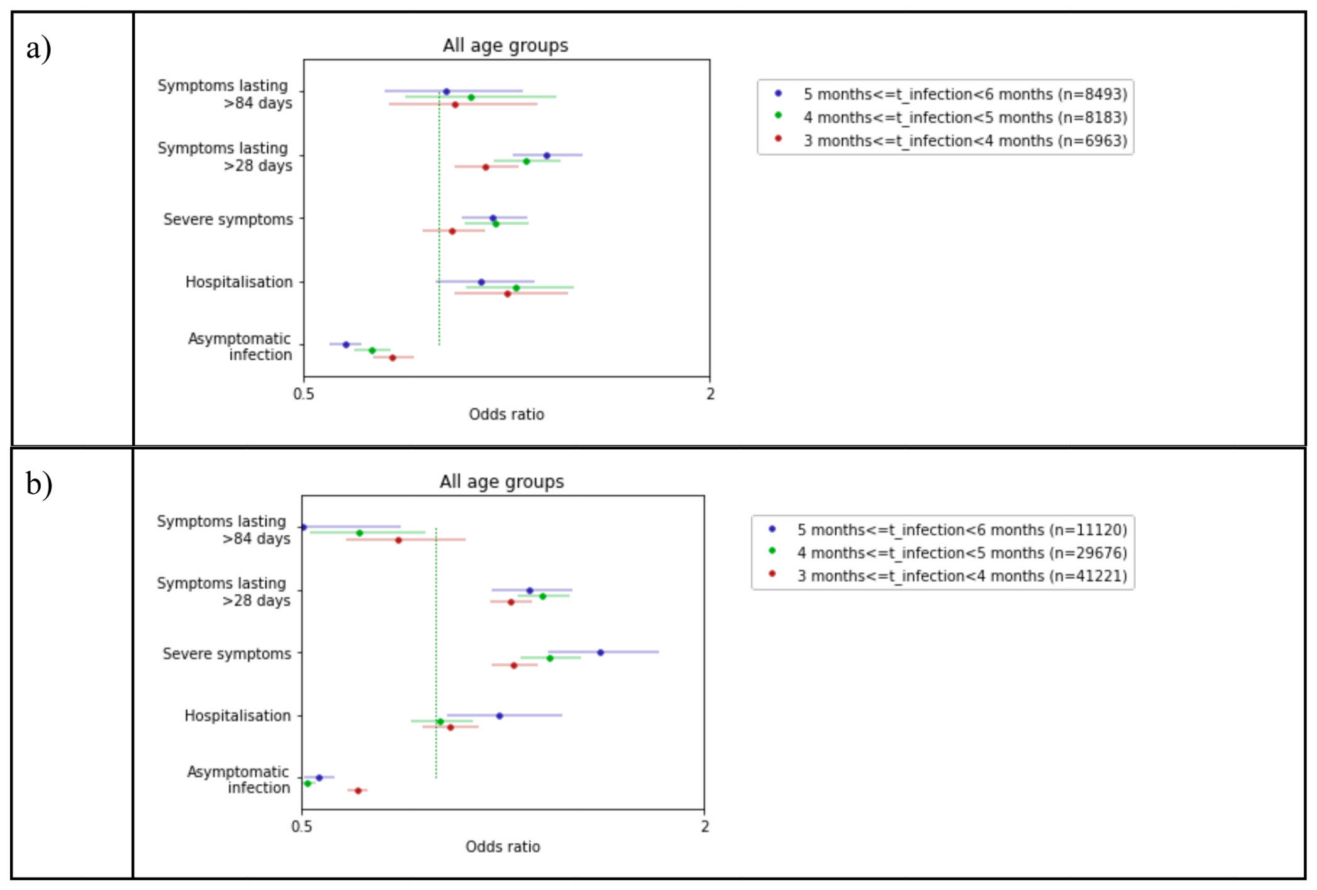
Odds ratio of asymptomatic infection, duration of symptoms ≥ 4 weeks, severe condition (two out of three among severe shortness of breath, fatigue, and fever), and hospitalization in individuals vaccinated with second dose during Delta period (a) and third dose during the Omicron period (b) and infected within the first 3 months from vaccination versus individuals vaccinated with the second dose and infected within 3-4, 4-5, and 5-6 months from vaccination adjusted by age, BMI, sex, frailty, and IMD.

During the Delta period, there was a significantly higher odds of symptoms lasting more than 4 weeks (from 1.17 to 1.40) and a decrease in the odds of asymptomatic infection (from 0.83 to 0.65) with increasing TSV. There was no difference in symptom severity with TSV > 4 months (Figure 5a, Supplementary Table 6). For the Omicron period, there was a clear VE waning for severe symptoms over the 6 months (OR increasing from 1.29 to 1.61), while for asymptomatic infection and duration ≥4 weeks the VE waning was significant only up to 5 months (OR decreasing from 0.71 to 0.56 for asymptomatic infections and increasing from 1.28 and 1.35 for LC). The associations for other severity indicators were not significant (Figure 5b, Supplementary Table 7).

**Figure 6:**
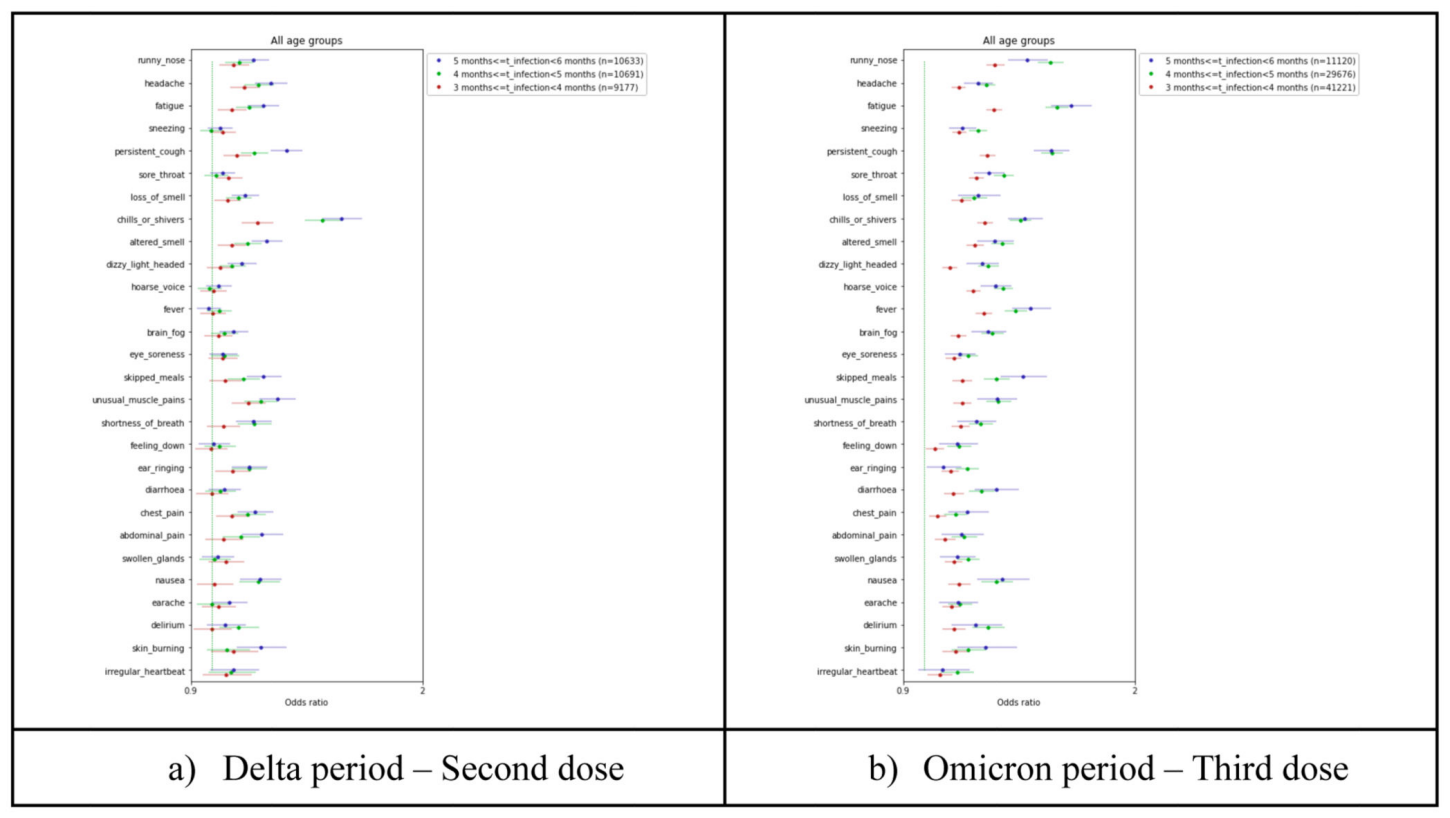
Odds Ratio of individual symptoms in individuals vaccinated with second dose during the Delta period (a) and third dose during the Omicron period (b) and infected within the first 3 months from vaccination versus individuals vaccinated with the second dose and infected within 3-4, 4-5, and 5-6 months from vaccination adjusted by age, BMI, sex, frailty, and IMD.

Regarding symptom profile, during both Delta and Omicron periods there was a clear waning of VE for almost all individual symptoms (Figures 6, Supplementary Tables 8 and 9). This was more evident during the Omicron period, although during this period no further waning of VE was observed when compared to 4<=TSV<=5 months and 5<=TSV<=6 months.

For both the analyses, we obtained the same results when stratified by age groups (see Supplementary Figures 3 and 4).

## Discussion

We present data on 155,967 and 56,236 community-based adults in the UK with reported test-confirmed SARS-CoV-2 infection after a third (booster) or second vaccination, respectively. During the period when Delta was the predominant COVID-19 variant, a third vaccination conferred reductions in disease severity and duration, and a higher proportion of infected individuals reporting asymptomatic illness. The observed protective effect of booster vaccination against long COVID is supported by findings from our previous study of over one million COVID Symptom Study participants, where long duration illness was reduced following a second vaccination dose, at a time when the Delta variant was predominant in the UK.^11^ In the current study, protective effects of booster vaccination were also apparent during the Omicron period, with a significant reduction in acute, functionally limiting symptoms, although not in hospitalization rate. The latter finding contrasts with that from a recent US-based study, which found that booster vaccines were effective in reducing COVID-19-associated Emergency Department and Urgent Care encounters and hospitalizations amongst immunocompetent adults, during a period when Omicron sublineages accounted for the majority of sequenced viral genomes in the US.^9^ Notably, the latter study looked at effects of bivalent mRNA vaccines, which were not approved as booster vaccines in the UK until August 2022.

This study adds to existing evidence on the symptom profile of infection following three or more vaccinations. During the period when Delta was most prevalent, following a third vaccination dose, infected individuals were less likely to report almost all individual symptoms, compared with those infected following only two vaccine doses. This is supported by findings from our previous study using COVID Symptom Study app data, which demonstrated lower rates of individual symptom reporting in vaccinated versus unvaccinated individuals.^11^ Also in keeping with our findings, a study of 1199 US essential and frontline workers demonstrated that recent vaccination with three mRNA vaccine doses was associated with attenuated symptoms and duration of illness.^28^ In the current study, during the Omicron period there was a reduction in symptoms commonly reported during the earlier stages of pandemic, such as fever and cough, as well as fatigue and headache and a reduction in fever and chills following third dose vaccination was also observed in the study of US frontline workers.^28^ This is important, since these symptoms have previously been shown to be predictive of more severe disease.^29^

During the Omicron period, there was increased reporting of certain “upper respiratory” symptoms in infected individuals following a third dose, including sneezing, runny nose, and hoarse voice. This is consistent with findings from our previous study, which found a higher reporting of sneezing following a second vaccine dose.^11^ This observation is also supported by a recent nationwide study, which found higher reporting of certain symptoms including runny nose and sore throat in fully vaccinated versus unvaccinated individuals^30^, as well as findings from a pre-print Japanese registry-based observational study which found that upper respiratory tract symptom burden was increased following vaccination.^31^ In that study, vaccination mitigated overall systemic symptoms and risk of severe disease, in keeping with our findings. One potential explanation for this could be “immune priming”. Vaccines work by priming the immune system, meaning that should the pathogen be encountered naturally, the immune system is able to react more quickly and effectively. Although upper respiratory symptoms have seldom been described in any studies looking at immune priming following vaccination, they are well documented in inflammatory conditions such as allergic rhinitis.^32^

During both Delta and Omicron periods, there was a trend towards waning of VE, with increased symptom reporting with time since vaccination following both second and third vaccine doses. This observation is supported by *in vitro* studies measuring antibody levels and immunogenicity against COVID-19 following two or three doses of vaccine, and by meta-analyses showing reduced effectiveness of vaccines against SARS-CoV-2 infection and symptomatic COVID-19 over time, although protection against severe disease remained high.^5, 23, 33, 34^ This study adds to existing evidence by suggesting waning of VE against symptomatic illness, and also against long duration illness (symptoms lasting ≥ 4 weeks). Of note, the proportion of infected individuals reporting ongoing symptoms ≥ 12 weeks was much lower than symptoms ≥ 4 weeks, and there were differences in trends in waning of vaccination protection against symptomatic illness with time since vaccination during Omicron between these groups. This supports the suggestion that ongoing symptomatic COVID-19 (4-12 weeks) and post-COVID-19 syndrome (≥12 weeks) may capture different disease states and highlights the need for ongoing standardization of definitions used for long COVID in clinical practice and research.^35^

Ongoing surveillance of predominant COVID-19 variants and effectiveness of current vaccines against emerging variants remain vital components of the coordinated international response to the ongoing challenges posed by COVID-19. Future research should consider effects of further booster vaccinations (such as the seasonal spring and autumn booster programmes) using bivalent vaccines approved in the UK and other comparator settings, on risk of infection, indicators of disease severity, and symptom profile.

### Strengths and Limitations

This study has several strengths. The mobile application data collection method facilitates collection of daily prospective information on a comprehensive set of symptoms, permitting analysis of both individual symptoms and overall illness duration (although note that necessary data censoring could have underestimated symptom duration for both cases and controls, as some individuals only had 2 weeks of logging after their positive test result). The matching of cases and controls on time since vaccination and timing of the post-vaccination test reduced the potential for bias, although small differences between the groups remained on matched variables. We acknowledge potential differences in logging by vaccinated individuals or those undertaking regular COVID-19 testing during the study period (for example, if required for work). Access to testing is a potential source of bias, although this was mitigated by only including reported infections up until April 1st, 2022, after which free tests were no longer universally available in the UK.

Our study has some limitations. We were unable to include an unvaccinated control group for comparison, as there were very few unvaccinated individuals enrolled at the time of the study. Our findings might not apply at all timepoints post-vaccination, to countries with different recommended vaccination types and schedules, or settings with different proportions of SARS-CoV-2 variants. Additionally, the data were self-reported; recording of comorbidities, test results, and vaccination status might not have been completely accurate and there might have been temporal gaps in reporting. We cannot exclude potential bias in self-reporting due to vaccination status – boosted individuals may have been less likely to report severe symptoms if they felt “protected” from the booster vaccination. Users of the COVID Symptom Study app are asked to log daily; therefore, if a participant reports on alternate days, the proportion of missing daily entries is 50%. However, given the typical duration of COVID-19 symptoms, the sampling frequencies in the COVID Symptom Study should have allowed good characterisation of infections. Only participants with at least 12 weeks of symptom reporting following the positive test were included; individuals with ongoing symptoms ≥ 12 weeks may have been affected by the long period of reporting required, and the size of the affected population underestimated. We only looked at waning of the protective effects of vaccination up to 6months, as there was limited data on participants after this date.

## Conclusions

This study suggests that a third monovalent vaccine dose reduces disease severity, duration and symptom burden in individuals infected following vaccination. Effects were more apparent during the Delta period, although disease severity was reduced during the Omicron period. Upper respiratory symptoms were more prevalent during the Omicron period, possibly due to immunological priming. Vaccine effectiveness waned after 3 months following most recent vaccination (for both third and second doses), with greater illness severity, duration, and symptom burden. Findings support ongoing efforts to promote booster vaccination, to reduce both illness severity amongst individuals at high-risk from COVID-19 and longer-term burden on health systems. Sharing knowledge on common symptoms in infection following booster vaccination is important to encourage informed vaccine uptake. Surveillance of emerging COVID variants and vaccine effectiveness remains vital in the coordinated international response to ongoing challenges posed by COVID-19.

## Supplementary Material

Supplementary Materials

## Figures and Tables

**Table 1:** Demographics and comorbidities of cases and controls for disease severity, duration and symptom profile analysis.

		Cases: users testing positive after 3rd dose				Controls: users testing positive after 2nd dose matched on, sex, age, BMI, and time since first vaccination		
		**Total**	**18-59 years**		**60+ years**	**Total**	**18-59 years**	**60+ years**
	**Delta**	1910	633		1277	1910	633	1277
	**Omicron**	7984	6214		1770	7641	5961	1680
**Baseline characteristics**								
**Female n(%)/Male n(%)**	**Delta**	1089(57.0) 821(43.0)		459(72.5) 174(27.5)	630(49.3) 647(50.7)	1110(58.2)^=^ 799(41.8)	477(70.5) ^=^ 156(29.5)	634(51.4) ^=^ 643(48.6)
	**Omicron**	4850(60.7) 3134(39.3)		3836(61.7) 2378(38.3)	1014(57.3) 756(42.7)	4646(58.2)* 2995(41.8)	3623(58.3)^=^ 2338(42.7)**	1023(57.8) ^=^ 657(42.2)
**Age (SD)**	**Delta**	64.0(12.8)		49.2(7.9)	71.4(7.0)	63.7(12.9) ^=^	48.5(7.7) ^=^	71.2(6.9)*
	**Omicron**	45.5(16.3)		38.9(11.3)	68.9(7.0)	44.3(17.7)*	37.3(13.0)*	68.9(7.0)^=^
**BMI (SD)**	**Delta**	26.8(6.5)		27.1(6.8)	26.7(6.3)	26.8(6.4) ^=^	27.1(6.7) ^=^	26.6(6.3)*
	**Omicron**	25.6(6.3)		25.3(6.2)	26.5(6.4)	25.3(6.6)*	24.9(6.6)*	26.4(6.5)^=^
**Comorbidities**								
**Cancer n(%)**	**Delta**	59(3.1)		8(1.3)	51(4.0)	42(2.2) ^=^	5(0.8)^=^	37(2.9) ^=^
	**Omicron**	55(0.7)		18(0.3)	37(2.1)	47(0.6)^=^	10(0.2)^=^	37(2.1)^=^
**Diabetes n(%)**	**Delta**	87(4.6)		21(3.3)	66(5.2)	109(5.7) ^=^	25(3.9)^=^	84(6.6) ^=^
	**Omicron**	125(1.6)		54(0.9)	71(4.0)	123(1.5)^=^	43(0.7)^=^	80(4.5)^=^
**Lung disease n(%)**	**Delta**	241(12.6)		88(13.9)	153(12.0)	208(10.9)^=^	80(12.6) ^=^	128(10.0) ^=^
	**Omicron**	644(8.1)		493(7.9)	151(8.5)	587(7.4)^=^	428(6.9)*	159(9.0)^=^
**Heart disease n(%)**	**Delta**	137(7.2)		13(2.1)	124(9.7)	133(7.0) ^=^	6(0.9)^=^	127(9.9) ^=^
	**Omicron**	142(1.8)		27(0.4)	115(6.5)	150(1.9)^=^	31(0.5)^=^	119(6.7)^=^
**Kidney disease n(%)**	**Delta**	25(1.3)		6(0.9)	19(1.5)	28(1.5) ^=^	7(1.1)^=^	21(1.6)^=^
	**Omicron**	42(0.5)		22(0.4)	20(1.1)	41(0.5)^=^	12(0.2)^=^	29(1.6)^=^
**Asthma n(%)**	**Delta**	286(15.0)		117(18.5)	169(13.2)	250(13.1) ^=^	98(15.5) ^=^	152(11.9) ^=^
	**Omicron**	951(11.9)		763(12.3)	188(10.6)	917(11.5)^=^	706(11.4)^=^	211(11.9)^=^
**Frailty n (%)**	**Delta**	156(8.2)		34(5.4)	122(9.6)	136(7.1) ^=^	20(3.2) ^=^	116(9.1) ^=^
	**Omicron**	193(2.4)		82(1.3)	111(6.3)	279(3.5)*	125(2.0)*	154(8.7)*
**Presence of at least one comorbidity**	**Delta**	556(29.1)		165(26.1)	391(30.6)	531(27.8) ^=^	141(22.3) ^=^	390(30.5) ^=^
	**Omicron**	1282(16.1)		881(14.2)	401(22.7)	1255(15.7)	820(13.2)^=^	435(24.6)^=^
**Level of Social Deprivation**								
**IMD [1-3] n(%)**	**Delta**	182(9.5)		75(11.8)	107(8.4)	176(9.2)^=^	77(12.2) ^=^	99(7.8) ^=^
	**Omicron**	1003(12.6)		854(13.7)	149(8.4)	1177(14.7)*	974(15.7)*	203(11.5)*
**IMD [4-7] n(%)**	**Delta**	697(36.5)		247(39.0)	450(35.2)	762(39.9) *	265(41.9) ^=^	497(38.9)^=^
	**Omicron**	3131(39.2)		2439(39.3)	692(39.1)	2965(37.1)*	2309(37.2)*	656(37.1)^=^
**IMD [8-10] n(%)**	**Delta**	938(49.1)		275(43.4)	663(51.9)	851(44.6)*	253(40.0) ^=^	598(46.8)*
	**Omicron**	3397(42.5)		2558(41.2)	839(47.4)	3253(40.7)*	2450(39.4)*	803(45.4)^=^

BMI=Body mass index; SD=Standard deviation; IMD=Index of Multiple Deprivation; IMD[1-3] indicates high social deprivation, IMD[4-7] intermediate, IMD[8-10] low; age is in years; comorbidity status=at least one comorbidity; for age and BMI the mean and standard deviation are provided, and for categorical variables the absolute value and percentages (%). */= Indicates statistically significant/no statistically significant difference when compared to the control population (Fisher’s p<0.05)

## References

[1] Department of Health and Social Care (2023). JCVI statement on the COVID-19 vaccination programme for 2023: 8 november 2022.

[2] Cheetham NJ, Kibble M, Wong A, Silverwood RJ, Knuppel A, Williams DM (2023). Antibody levels following vaccination against SARS-CoV-2: associations with post-vaccination infection and risk factors in two UK longitudinal studies. Elife.

[3] Lee J, Lee DG, Jung J, Ryu JH, Shin S, Cho SY (2022). Comprehensive assessment of SARS-CoV-2 antibodies against various antigenic epitopes after naive COVID-19 infection and vaccination (BNT162b2 or ChAdOx1 nCoV-19). Front Immunol.

[4] Cheng SMS, Mok CKP, Leung YWY, Ng SS, Chan KCK, Ko FW (2022). Neutralizing antibodies against the SARS-CoV-2 Omicron variant BA.1 following homologous and heterologous CoronaVac or BNT162b2 vaccination. Nat Med.

[5] Zhou R, Liu N, Li X, Peng Q, Yiu CK, Huang H (2022). Three-dose vaccination-induced immune responses protect against SARS-CoV-2 Omicron BA.2: A population-based study in Hong Kong. Lancet Reg Health West Pac.

[6] Gruell H, Vanshylla K, Tober-Lau P, Hillus D, Schommers P, Lehmann C (2022). mRNA booster immunization elicits potent neutralizing serum activity against the SARS-CoV-2 Omicron variant. Nat Med.

[7] Glatman-Freedman A, Bromberg M, Hershkovitz Y, Sefty H, Kaufman Z, Dichtiar R (2022). Effectiveness of BNT162b2 Vaccine Booster against SARS-CoV-2 Infection and Breakthrough Complications, Israel. Emerg Infect Dis.

[8] Andrews N, Stowe J, Kirsebom F, Toffa S, Sachdeva R, Gower C (2022). Effectiveness of COVID-19 booster vaccines against COVID-19-related symptoms, hospitalization and death in England. Nat Med.

[9] Tenforde MW, Weber ZA, Natarajan K, Klein NP, Kharbanda AB, Stenehjem E (2022). Early Estimates of Bivalent mRNA Vaccine Effectiveness in Preventing COVID-19-Associated Emergency Department or Urgent Care Encounters and Hospitalizations Among Immunocompetent Adults - VISION Network, Nine States, September-November 2022. MMWR Morb Mortal Wkly Rep.

[10] Kelly JD, Leonard S, Hoggatt KJ, Boscardin WJ, Lum EN, Moss-Vazquez TA (2022). Incidence of Severe COVID-19 Illness Following Vaccination and Booster With BNT162b2, mRNA-1273, and Ad26.COV2.S Vaccines. Jama.

[11] Antonelli M, Penfold RS, Merino J, Sudre CH, Molteni E, Berry S (2022). Risk factors and disease profile of post-vaccination SARS-CoV-2 infection in UK users of the COVID Symptom Study app: a prospective, community-based, nested, case-control study. Lancet Infect Dis.

[12] Horne EMF, Hulme WJ, Keogh RH, Palmer TM, Williamson EJ, Parker EPK (2022). Waning effectiveness of BNT162b2 and ChAdOx1 covid-19 vaccines over six months since second dose: OpenSAFELY cohort study using linked electronic health records. Bmj.

[13] Wei Y, Jia KM, Zhao S, Hung CT, Mok CKP, Poon PKM (2023). Estimation of Vaccine Effectiveness of CoronaVac and BNT162b2 Against Severe Outcomes Over Time Among Patients With SARS-CoV-2 Omicron. JAMA Netw Open.

[14] Andrews N, Stowe J, Kirsebom F, Toffa S, Rickeard T, Gallagher E (2022). Covid-19 Vaccine Effectiveness against the Omicron (B.1.1.529) Variant. N Engl J Med.

[15] Menni C, May A, Polidori L, Louca P, Wolf J, Capdevila J (2022). COVID-19 vaccine waning and effectiveness and side-effects of boosters: a prospective community study from the ZOE COVID Study. Lancet Infect Dis.

[16] Link-Gelles R, Levy ME, Natarajan K, Reese SE, Naleway AL, Grannis SJ (2023). Estimation of COVID-19 mRNA Vaccine Effectiveness and COVID-19 Illness and Severity by Vaccination Status During Omicron BA.4 and BA.5 Sublineage Periods. JAMA Netw Open.

[17] Cerqueira-Silva T, Shah SA, Robertson C, Sanchez M, Katikireddi SV, de Araujo Oliveira V (2023). Effectiveness of mRNA boosters after homologous primary series with BNT162b2 or ChAdOx1 against symptomatic infection and severe COVID-19 in Brazil and Scotland: A test-negative design case-control study. PLoS Med.

[18] Brazete C, Brazete J, Alves F, Aguiar A, Gonçalves AM, Cardoso M (2023). COVID-19 vaccines effectiveness against symptomatic disease and severe outcomes, 2021-2022: a test-negative case-control study. Public Health.

[19] Patalon T, Saciuk Y, Peretz A, Perez G, Lurie Y, Maor Y (2022). Waning effectiveness of the third dose of the BNT162b2 mRNA COVID-19 vaccine. Nat Commun.

[20] Menni C, Valdes AM, Freidin MB, Sudre CH, Nguyen LH, Drew DA (2020). Real-time tracking of self-reported symptoms to predict potential COVID-19. Nat Med.

[21] Spiel C, Lapka D, Gradinger P, Zodlhofer EM, Reimann R, Schober B (2008). A Euclidean distance-based matching procedure for nonrandomized comparison studies. European Psychologist.

[22] Centers for Disease Control and Prevention Symptoms of COVID-19.

[23] Cheetham NJ, Kibble M, Wong A, Silverwood RJ, Knuppel A, Williams DM (2022). Antibody levels following vaccination against SARS-CoV-2: associations with post-vaccination infection and risk factors. medRxiv.

[24] Ferdinands JM, Rao S, Dixon BE, Mitchell PK, DeSilva MB, Irving SA (2022). Waning of vaccine effectiveness against moderate and severe covid-19 among adults in the US from the VISION network: test negative, case-control study. Bmj.

[25] Murray B, Kerfoot E, Chen L, Deng J, Graham MS, Sudre CH (2021). Accessible data curation and analytics for international-scale citizen science datasets. Scientific Data.

[26] Zazzara MB, Penfold RS, Roberts AL, Lee KA, Dooley H, Sudre CH (2021). Probable delirium is a presenting symptom of COVID-19 in frail, older adults: a cohort study of 322 hospitalised and 535 community-based older adults. Age Ageing.

[27] Raîche M, Hébert R, Dubois M-F (2008). PRISMA-7: a case-finding tool to identify older adults with moderate to severe disabilities. Archives of gerontology and geriatrics.

[28] Thompson MG, Yoon SK, Naleway AL, Meece J, Fabrizio TP, Caban-Martinez AJ (2022). Association of mRNA Vaccination With Clinical and Virologic Features of COVID-19 Among US Essential and Frontline Workers. Jama.

[29] Sudre CH, Lee KA, Lochlainn MN, Varsavsky T, Murray B, Graham MS (2021). Symptom clusters in COVID-19: A potential clinical prediction tool from the COVID Symptom Study app. Sci Adv.

[30] Spiliopoulos L, Sørensen AIV, Bager P, Nielsen NM, Hansen JV, Koch A (2022). Post-acute symptoms four months after SARS-CoV-2 infection during the Omicron period: a nationwide Danish questionnaire study. medRxiv.

[31] Nakakubo S, Kishida N, Okuda K, Kamada K, Iwama M, Suzuki M (2023). Associations of COVID-19 Symptoms with Omicron Subvariants BA.2 and BA.5, Host Status, and Clinical Outcomes: A Registry-Based Observational Study in Sapporo, Japan. medRxiv.

[32] Eifan AO, Durham SR (2016). Pathogenesis of rhinitis. Clin Exp Allergy.

[33] Ssentongo P, Ssentongo AE, Voleti N, Groff D, Sun A, Ba DM (2022). SARS-CoV-2 vaccine effectiveness against infection, symptomatic and severe COVID-19: a systematic review and meta-analysis. BMC Infect Dis.

[34] Feikin DR, Higdon MM, Abu-Raddad LJ, Andrews N, Araos R, Goldberg Y (2022). Duration of effectiveness of vaccines against SARS-CoV-2 infection and COVID-19 disease: results of a systematic review and meta-regression. Lancet.

[35] Shah W, Heightman M, O'Brien S (2021). UK guidelines for managing long-term effects of COVID-19. Lancet (London, England).

