# Supplementary material for: SARS-CoV-2 infection following booster vaccination: illness and symptom profile in a prospective, observational community-based case-control study
Source: J Infect. Author manuscript; Available in PMC 2025 Oct 24. (PMC7618294; doi:10.1016/j.jinf.2023.08.009)
Supplement: Supplementary Materials [file EMS209510-suppement-Supplementary_Materials.pdf]

Supplementary Table 1. List of all self-reported symptoms and corresponding questions used in the reporting app.

Symptoms	Questions
fever	Do you have a fever or feel too hot?
chills_or_shivers	Do you feel chills or shivers (feel too cold)?
persistent_cough	Persistent cough (coughing a lot for more than an hour or 3 or more coughing episodes in 24 hours)
fatigue	Are you experiencing unusual fatigue?
shortness_of_breath	Shortness of breath or trouble breathing
loss_of_smell	Loss of smell / taste
hoarse_voice	Unusually hoarse voice
chest_pain	Unusual chest pain or tightness in your chest
abdominal_pain	Unusual abdominal pain or stomach ache
diarrhoea	Diarrhoea
delirium	Confusion, disorientation or drowsiness
eye_soreness	Do your eyes have any unusual eye-soreness or discomfort (e.g. light sensitivity, excessive tears, or pink/red eye)?
skipped_meals	Skipping meals
headache	Headache
nausea	Nausea or vomiting
dizzy_light_headed	Dizziness or light-headedness
sore_throat	Sore or painful throat
unusual_muscle_pains	Unusual strong muscle pains or aches
red_welts_on_face_or_lips	Raised, red, itchy welts on the skin or sudden swelling of the face or lips
blisters_on_feet	Red/purple sores or blisters on your feet, including your toes
typical_hayfever	Increase in your usual allergy symptoms
rash	Rash on your arms or torso
skin_burning	Strange, unpleasant sensations in your skin like “pins & needles” or burning
hair_loss	Unusual hair loss
feeling_down	Feeling down, depressed or hopeless
brain_fog	Loss of concentration or memory (brain fog)
runny_nose	Runny nose
sneezing	Sneezing more than usual
earache	Earache
ear_ringing	Ringing in your ears
swollen_glands	Swollen neck glands
irregular_heartbeat	Unusually fast or irregular heartbeat (palpitations)

Supplementary Table 2. Univariate analysis assessing the probability of asymptomatic infection, severe disease (two out of three among severe shortness of breath, fatigue, and fever), hospitalization and duration of symptoms ≥ 4/12weeks in app participants following booster vaccination, adjusted by age, BMI, sex, frailty, and IMD for the Delta period.

	All age groups			Younger adults (18-59 years)			Older adults (60+ years)		
	OR	95% CI	p-value	OR	95% CI	p-value	OR	95% CI	p-value
Symptoms lasting ≥12 weeks	0.83	[0.50-1.36]	0.454	0.62	[0.22-1.73]	0.361	0.91	[0.51-1.62]	0.747
Symptoms lasting ≥4 weeks	0.56	[0.44-0.70]	<0.0001	0.70	[0.44-1.10]	0.124	0.52	[0.40-0.68]	<0.0001
Severe symptoms	0.36	[0.27-0.49]	<0.0001	0.33	[0.21-0.51]	<0.0001	0.38	[0.26-0.56]	<0.0001
hospitalization	0.55	[0.39-0.75]	<0.0001	0.53	[0.26-1.05]	0.07	0.56	[0.38-0.82]	0.003
Asymptomatic infections	3.45	[2.86-4.16]	<0.0001	3.62	[2.48-5.28]	<0.0001	3.45	[2.77-4.29]	<0.0001

OR = Odds ratio; CI = Confidence interval;

Supplementary Table 3. Univariate analysis assessing the probability of asymptomatic infection, severe disease (two out of three among severe shortness of breath, fatigue, and fever), hospitalization and duration of symptoms ≥ 4/12weeks in app participants following booster vaccination, adjusted by age, BMI, sex, frailty, and IMD for the Omicron period.

	All age groups			Younger adults (18-59 years)			Older adults (60+ years)		
	OR	95% CI	p-value	OR	95% CI	p-value	OR	95% CI	p-value
Symptoms lasting ≥12 weeks	0.77	[0.39-1.52]	0.448	0.73	[0.29-1.86]	0.511	NaN	[NaN-NaN]	NaN
Symptoms lasting ≥4 weeks	1.01	[0.85-1.19]	0.948	0.91	[0.73-1.12]	0.368	1.10	[0.83-1.45]	0.001
Severe symptoms	0.48	[0.42-0.55]	<0.0001	0.46	[0.39-0.53]	<0.0001	0.57	[0.41-0.79]	0.258
Hospitalization	0.86	[0.68-1.08]	0.189	0.88	[0.66-1.16]	0.353	0.79	[0.52-1.19]	0.063
Asymptomatic infections	0.99	[0.89-1.09]	0.771	1.07	[0.95-1.20]	0.259	0.82	[0.66-1.01]	0.508

OR = Odds ratio; CI = Confidence interval;

Supplementary Table 4. Univariate analysis assessing the probability of experiencing each symptom in app participants following booster vaccination, adjusted by age, BMI, sex, frailty, and IMD for the Delta period.

	All age groups			Younger adults (18-59 years)			Older adults (60+ years)		
	OR	95% CI	p-value	OR	95% CI	p-value	OR	95% CI	p-value
runny_nose	0.54	[0.47-0.61]	<0.0001	0.48	[0.38-0.62]	<0.0001	0.55	[0.47-0.65]	<0.0001
headache	0.49	[0.43-0.56]	<0.0001	0.38	[0.29-0.48]	<0.0001	0.54	[0.46-0.63]	<0.0001
fatigue	0.40	[0.35-0.46]	<0.0001	0.43	[0.34-0.54]	<0.0001	0.39	[0.33-0.46]	<0.0001
sneezing	0.68	[0.60-0.78]	<0.0001	0.67	[0.54-0.85]	0.001	0.68	[0.58-0.80]	<0.0001
persistent_cough	0.36	[0.31-0.41]	<0.0001	0.30	[0.24-0.38]	<0.0001	0.38	[0.32-0.45]	<0.0001
sore_throat	0.66	[0.57-0.75]	<0.0001	0.65	[0.52-0.82]	<0.0001	0.65	[0.55-0.77]	<0.0001
loss_of_smell	0.41	[0.36-0.47]	<0.0001	0.36	[0.28-0.45]	<0.0001	0.44	[0.36-0.52]	<0.0001
chills_or_shivers	0.40	[0.35-0.46]	<0.0001	0.32	[0.25-0.41]	<0.0001	0.44	[0.37-0.52]	<0.0001
altered_smell	0.47	[0.41-0.54]	<0.0001	0.46	[0.36-0.58]	<0.0001	0.47	[0.39-0.57]	<0.0001
dizzy_light_headed	0.51	[0.44-0.59]	<0.0001	0.48	[0.38-0.62]	<0.0001	0.51	[0.42-0.62]	<0.0001
hoarse_voice	0.54	[0.46-0.62]	<0.0001	0.39	[0.30-0.50]	<0.0001	0.63	[0.52-0.76]	<0.0001
fever	0.36	[0.31-0.42]	<0.0001	0.33	[0.25-0.42]	<0.0001	0.37	[0.30-0.45]	<0.0001
eye_soreness	0.69	[0.59-0.80]	<0.0001	0.70	[0.54-0.89]	0.005	0.67	[0.55-0.81]	<0.0001
brain_fog	0.54	[0.46-0.63]	<0.0001	0.60	[0.47-0.77]	<0.0001	0.50	[0.41-0.61]	<0.0001
skipped_meals	0.37	[0.31-0.44]	<0.0001	0.45	[0.34-0.60]	<0.0001	0.32	[0.26-0.40]	<0.0001
unusual_muscle_pains	0.44	[0.37-0.52]	<0.0001	0.34	[0.26-0.45]	<0.0001	0.51	[0.41-0.64]	<0.0001
shortness_of_breath	0.54	[0.45-0.64]	<0.0001	0.58	[0.44-0.77]	<0.0001	0.50	[0.40-0.64]	<0.0001
feeling_down	0.68	[0.57-0.82]	<0.0001	0.84	[0.63-1.14]	0.265	0.60	[0.47-0.76]	<0.0001
chest_pain	0.58	[0.48-0.70]	0.002	0.52	[0.39-0.69]	<0.0001	0.63	[0.49-0.80]	0.01
diarrhoea	0.54	[0.45-0.66]	<0.0001	0.66	[0.48-0.91]	0.012	0.48	[0.38-0.61]	<0.0001
ear_ringing	0.75	[0.62-0.90]	<0.0001	0.71	[0.53-0.96]	0.025	0.76	[0.60-0.96]	0.021
abdominal_pain	0.67	[0.55-0.82]	<0.0001	0.70	[0.51-0.94]	0.019	0.64	[0.50-0.83]	0.001
nausea	0.43	[0.35-0.53]	<0.0001	0.42	[0.31-0.58]	<0.0001	0.42	[0.32-0.55]	<0.0001
swollen_glands	0.65	[0.53-0.79]	<0.0001	0.65	[0.49-0.87]	0.003	0.63	[0.47-0.83]	0.001
earache	0.59	[0.48-0.72]	<0.0001	0.59	[0.44-0.79]	0.001	0.58	[0.43-0.77]	<0.0001

delirium	0.47	[0.37-0.59]	<0.0001	0.44	[0.30-0.64]	<0.0001	0.49	[0.36-0.66]	<0.0001
skin_burning	0.47	[0.36-0.61]	<0.0001	0.56	[0.38-0.83]	0.004	0.39	[0.27-0.56]	<0.0001
irregular_heartbeat	0.62	[0.48-0.80]	<0.0001	0.56	[0.38-0.82]	0.003	0.65	[0.46-0.92]	0.016

OR = Odds ratio; CI = Confidence interval

Supplementary Table 5. Univariate analysis assessing the probability of experiencing each symptom in app participants following booster vaccination, adjusted by age, BMI, sex, frailty, and IMD for the Omicron period.

	All age groups			Younger adults (18-59 years)			Older adults (60+ years)		
	OR	95% CI	p-value	OR	95% CI	p-value	OR	95% CI	p-value
runny_nose	1.26	[1.18-1.35]	<0.0001	1.23	[1.15-1.34]	<0.0001	1.30	[1.12-1.51]	<0.0001
headache	0.88	[0.83-0.94]	<0.0001	0.82	[0.76-0.88]	<0.0001	1.06	[0.93-1.22]	0.37
fatigue	0.86	[0.81-0.92]	<0.0001	0.88	[0.80-0.92]	<0.0001	0.83	[0.73-0.96]	0.009
sneezing	1.40	[1.32-1.50]	<0.0001	1.39	[1.30-1.51]	<0.0001	1.37	[1.20-1.57]	<0.0001
persistent_cough	0.93	[0.87-0.99]	0.031	0.90	[0.84-0.97]	0.007	1.01	[0.88-1.15]	0.937
sore_throat	1.17	[1.10-1.25]	<0.0001	1.19	[1.09-1.26]	<0.0001	1.14	[0.99-1.30]	0.061
loss_of_smell	0.76	[0.69-0.83]	<0.0001	0.73	[0.64-0.79]	<0.0001	0.92	[0.75-1.13]	0.423
chills_or_shivers	0.59	[0.55-0.63]	<0.0001	0.55	[0.50-0.58]	<0.0001	0.75	[0.65-0.87]	<0.0001
altered_smell	0.76	[0.71-0.83]	<0.0001	0.75	[0.67-0.80]	<0.0001	0.85	[0.72-1.01]	0.061
dizzy_light_headed	0.81	[0.76-0.88]	<0.0001	0.78	[0.71-0.84]	<0.0001	0.94	[0.80-1.10]	0.441
hoarse_voice	1.13	[1.06-1.21]	<0.0001	1.12	[1.03-1.20]	0.008	1.15	[1.01-1.32]	0.038
fever	0.67	[0.62-0.72]	<0.0001	0.61	[0.55-0.65]	<0.0001	0.93	[0.80-1.08]	0.317
eye_soreness	0.96	[0.88-1.04]	0.292	0.92	[0.83-0.99]	0.037	1.10	[0.94-1.30]	0.245
brain_fog	1.09	[1.01-1.17]	0.034	1.15	[1.04-1.23]	0.006	0.89	[0.75-1.05]	0.167
skipped_meals	0.58	[0.53-0.64]	<0.0001	0.54	[0.47-0.58]	<0.0001	0.77	[0.64-0.92]	0.003
unusual_muscle_pains	0.71	[0.66-0.77]	<0.0001	0.72	[0.64-0.77]	<0.0001	0.68	[0.57-0.81]	<0.0001
shortness_of_breath	0.95	[0.87-1.04]	0.272	0.98	[0.89-1.08]	0.68	0.80	[0.66-0.98]	0.029
feeling_down	1.03	[0.94-1.13]	0.558	0.97	[0.97-1.19]	0.169	0.85	[0.70-1.04]	0.109
chest_pain	0.97	[0.89-1.06]	0.448	0.87	[0.87-1.05]	0.373	0.94	[0.77-1.15]	0.546
diarrhoea	0.79	[0.72-0.87]	<0.0001	0.85	[0.73-0.91]	0.003	0.68	[0.56-0.83]	<0.0001

ear_ringing	0.88	[0.80-0.96]	0.006	1.08	[0.76-0.94]	0.002	0.95	[0.79-1.16]	0.631
abdominal_pain	0.81	[0.73-0.89]	<0.0001	0.79	[0.70-0.87]	<0.0001	0.89	[0.72-1.10]	0.3
nausea	0.64	[0.57-0.70]	<0.0001	0.67	[0.58-0.72]	<0.0001	0.58	[0.46-0.73]	<0.0001
swollen_glands	1.05	[0.96-1.14]	0.273	1.04	[0.95-1.14]	0.396	1.00	[0.82-1.21]	0.978
earache	0.92	[0.84-1.00]	0.058	0.86	[0.77-0.94]	0.002	1.15	[0.94-1.41]	0.166
delirium	0.82	[0.74-0.91]	<0.0001	0.88	[0.78-0.98]	0.022	0.61	[0.48-0.77]	<0.0001
skin_burning	0.65	[0.57-0.75]	<0.0001	0.65	[0.56-0.77]	<0.0001	0.62	[0.46-0.81]	0.001
irregular_heartbeat	0.75	[0.66-0.85]	<0.0001	0.71	[0.62-0.83]	<0.0001	0.86	[0.64-1.14]	0.289
OR = Odds ratio; CI = Confidence interval									

Supplementary Table 6. Odds Ratio of asymptomatic infection, severe disease (two out of three among severe shortness of breath, fatigue, and fever), hospitalization and duration of symptoms ≥ 4/12weeks in individuals with second dose and infected within 3 months compared to individuals with time since vaccination (TSV) between 3-4, 4-5, 5-6 months for the Delta period.

		All age groups			Younger adults (18-59 years)			Older adults (60+ years)		
		OR	95% CI	p-value	OR	95% CI	p-value	OR	95% CI	p-value
Symptoms lasting ≥12 weeks	3-4 months	1.06	[0.82-1.37]	0.674	0.99	[0.66-1.49]	0.967	0.99	[0.66-1.49]	0.967
	4-5 months	1.12	[0.87-1.43]	0.369	1.08	[0.73-1.61]	0.693	1.08	[0.73-1.61]	0.693
	5-6 months	1.03	[0.80-1.31]	0.84	0.835	[0.53-1.35]	0.485	0.85	[0.53-1.35]	0.485
Symptoms lasting ≥4 weeks	3-4 months	1.17	[1.06-1.29]	0.002	1.15	[1.00-1.32]	0.05	1.20	[1.04-1.39]	0.012
	4-5 months	1.32	[1.21-1.45]	<0.0001	1.35	[1.18-1.54]	<0.0001	1.34	[1.17-1.53]	<0.0001
	5-6 months	1.40	[1.28-1.53]	<0.0001	1.42	[1.23-1.63]	<0.0001	1.43	[1.26-1.62]	<0.0001
Severe symptoms	3-4 months	1.05	[0.94-1.17]	0.377	1.00	[0.87-1.14]	0.948	1.15	[0.96-1.36]	0.121
	4-5 months	1.21	[1.10-1.33]	<0.0001	1.21	[1.07-1.38]	0.003	1.25	[1.07-1.46]	0.004
	5-6 months	1.20	[1.09-1.33]	<0.0001	1.27	[1.11-1.46]	<0.0001	1.20	[1.04-1.39]	0.014
hospitalization	3-4 months	1.25	[1.06-1.48]	0.008	1.30	[1.03-1.65]	0.03	1.21	[0.95-1.53]	0.118
	4-5 months	1.29	[1.10-1.50]	0.002	1.31	[1.04-1.65]	0.02	1.26	[1.02-1.56]	0.031
	5-6 months	1.16	[0.99-1.36]	0.068	1.17	[0.91-1.52]	0.22	1.15	[0.94-1.41]	0.178
Asymptomatic infections	3-4 months	0.83	[0.76-0.91]	<0.0001	0.77	[0.67-0.88]	<0.0001	0.87	[0.76-0.99]	0.034

	4-5 months	0.75	[0.69-0.82]	<0.0001	0.66	[0.58-0.76]	<0.0001	0.80	[0.71-0.90]	<0.0001
	5-6 months	0.65	[0.60-0.72]	<0.0001	0.75	[0.65-0.87]	<0.0001	0.56	[0.50-0.63]	<0.0001

OR = Odds ratio; CI = Confidence interval;

Supplementary Table 7. Odds Ratio of asymptomatic infection, severe disease (two out of three among severe shortness of breath, fatigue, and fever), hospitalization and duration of symptoms ≥ 4/12 weeks in individuals with third dose and infected within 3 months compared to individuals with time since vaccination (TSV) between 3-4, 4-5, 5-6 months for the Omicron period.

		All age groups			Younger adults (18-59 years)			Older adults (60+ years)		
		OR	95% CI	p-value	OR	95% CI	p-value	OR	95% CI	p-value
Symptoms lasting ≥12 weeks	3-4 months	0.86	[0.67-1.12]	0.259	1.04	[0.59-1.83]	0.903	0.82	[0.62-1.10]	0.180
	4-5 months	0.72	[0.53-0.96]	0.028	0.70	[0.29-1.72]	0.438	0.72	[0.52-0.99]	0.042
	5-6 months	0.51	[0.29-0.87]	0.014	0.89	[0.22-3.59]	0.867	0.48	[0.26-0.87]	0.015
Symptoms lasting ≥4 weeks	3-4 months	1.28	[1.21-1.36]	<0.0001	1.20	[1.08-1.34]	0.001	1.32	[1.22-1.42]	<0.0001
	4-5 months	1.40	[1.31-1.50]	<0.0001	1.24	[1.03-1.49]	0.023	1.45	[1.34-1.56]	<0.0001
	5-6 months	1.35	[1.21-1.51]	<0.0001	1.11	[0.84-1.48]	0.465	1.42	[1.25-1.60]	<0.0001
Severe symptoms	3-4 months	1.29	[1.21-1.38]	<0.0001	1.31	[1.19-1.44]	<0.0001	1.27	[1.16-1.39]	<0.0001
	4-5 months	1.42	[1.32-1.54]	<0.0001	1.34	[1.13-1.59]	0.001	1.49	[1.36-1.63]	<0.0001
	5-6 months	1.61	[1.42-1.83]	<0.0001	1.66	[1.28-2.16]	<0.0001	1.62	[1.40-1.88]	<0.0001
hospitalization	3-4 months	1.05	[0.95-1.16]	0.331	1.30	[1.09-1.56]	0.004	0.95	[0.84-1.08]	0.429
	4-5 months	1.02	[0.91-1.14]	0.763	1.44	[1.06-1.97]	0.021	0.95	[0.84-1.07]	0.378
	5-6 months	1.24	[1.04-1.47]	0.015	2.09	[1.29-3.40]	0.003	1.12	[0.93-1.35]	0.249
Asymptomatic infections	3-4 months	0.71	[0.67-0.5]	<0.0001	0.77	[0.69-0.85]	<0.0001	0.69	[0.65-0.73]	<0.0001
	4-5 months	0.52	[0.49-0.5]	<0.0001	0.97	[0.80-1.18]	0.759	0.48	[0.45-0.51]	<0.0001
	5-6 months	0.56	[0.51-0.62]	<0.0001	0.99	[0.72-1.36]	0.948	0.53	[0.47-0.58]	<0.0001

OR = Odds ratio; CI = Confidence interval;

Supplementary Table 8. Odds Ratio of experiencing each symptom in individuals with second dose and infected within 3 months, compared to individuals with time since vaccination (TSV) between 3-4, 4-5, 5-6 months for the Delta period.

	All age groups			Younger adults (18-59 years)			Older adults (60+ years)		
	OR	95% CI	p-value	OR	95% CI	p-value	OR	95% CI	p-value

runny_nose	1.10	[1.04-1.18]	0.002	1.12	[1.03-1.22]	0.011	1.10	[1.00-1.21]	0.043
	1.13	[1.06-1.20]	<0.0001	1.17	[1.08-1.28]	<0.0001	1.11	[1.02-1.21]	0.015
	1.20	[1.13-1.27]	<0.0001	1.15	[1.05-1.26]	0.003	1.27	[1.17-1.38]	<0.0001
headache	1.16	[1.09-1.23]	<0.0001	1.24	[1.14-1.35]	<0.0001	1.10	[1.00-1.20]	0.053
	1.22	[1.15-1.29]	<0.0001	1.30	[1.19-1.41]	<0.0001	1.21	[1.12-1.32]	<0.0001
	1.28	[1.20-1.36]	<0.0001	1.35	[1.23-1.48]	<0.0001	1.31	[1.21-1.42]	<0.0001
fatigue	1.09	[1.03-1.16]	0.004	1.11	[1.02-1.20]	0.016	1.09	[0.99-1.19]	0.075
	1.18	[1.11-1.25]	<0.0001	1.19	[1.10-1.29]	<0.0001	1.20	[1.10-1.30]	<0.0001
	1.24	[1.17-1.32]	<0.0001	1.27	[1.16-1.39]	<0.0001	1.28	[1.18-1.39]	<0.0001
sneezing	1.05	[0.99-1.12]	0.098	1.02	[0.94-1.11]	0.614	1.11	[1.01-1.21]	0.029
	1.00	[0.94-1.05]	0.931	0.98	[0.91-1.06]	0.660	1.04	[0.96-1.13]	0.370
	1.04	[0.98-1.10]	0.198	0.96	[0.88-1.04]	0.316	1.14	[1.05-1.23]	0.001
persistent_cough	1.12	[1.06-1.19]	<0.0001	1.14	[1.05-1.23]	0.002	1.11	[1.01-1.21]	0.023
	1.20	[1.14-1.27]	<0.0001	1.26	[1.17-1.35]	<0.0001	1.18	[1.09-1.28]	<0.0001
	1.35	[1.28-1.43]	<0.0001	1.39	[1.28-1.51]	<0.0001	1.38	[1.28-1.48]	<0.0001
sore_throat	1.08	[1.02-1.15]	0.009	1.09	[1.01-1.18]	0.023	1.08	[0.99-1.19]	0.084
	1.02	[0.97-1.08]	0.453	1.01	[0.94-1.09]	0.780	1.06	[0.97-1.15]	0.190
	1.05	[0.99-1.11]	0.083	1.04	[0.96-1.13]	0.332	1.08	[1.00-1.17]	0.040
loss_of_smell	1.07	[1.01-1.14]	0.018	1.12	[1.04-1.21]	0.005	1.03	[0.94-1.13]	0.480
	1.13	[1.07-1.19]	<0.0001	1.15	[1.07-1.24]	<0.0001	1.14	[1.05-1.24]	0.002
	1.16	[1.09-1.23]	<0.0001	1.15	[1.06-1.26]	0.001	1.24	[1.15-1.34]	<0.0001
chills_or_shivers	1.22	[1.14-1.29]	<0.0001	1.19	[1.10-1.29]	<0.0001	1.26	[1.14-1.38]	<0.0001
	1.53	[1.44-1.61]	<0.0001	1.54	[1.43-1.67]	<0.0001	1.57	[1.44-1.71]	<0.0001
	1.62	[1.53-1.71]	<0.0001	1.63	[1.50-1.78]	<0.0001	1.69	[1.56-1.83]	<0.0001
altered_smell	1.09	[1.03-1.16]	0.004	1.09	[1.01-1.19]	0.028	1.10	[1.00-1.21]	0.045
	1.17	[1.11-1.24]	<0.0001	1.16	[1.08-1.25]	<0.0001	1.21	[1.11-1.32]	<0.0001
	1.26	[1.19-1.34]	<0.0001	1.23	[1.13-1.34]	<0.0001	1.34	[1.24-1.45]	<0.0001
dizzy_light_headed	1.04	[0.98-1.11]	0.219	1.08	[0.99-1.17]	0.085	1.00	[0.91-1.10]	0.981
	1.10	[1.03-1.16]	0.002	1.15	[1.06-1.24]	<0.0001	1.05	[0.96-1.14]	0.297
	1.14	[1.08-1.21]	<0.0001	1.23	[1.13-1.34]	<0.0001	1.11	[1.03-1.21]	0.010
hoarse_voice	1.01	[0.95-1.07]	0.821	1.05	[0.97-1.14]	0.239	0.97	[0.88-1.06]	0.486

	0.99	[0.93-1.05]	0.699	0.98	[0.90-1.06]	0.535	1.03	[0.94-1.12]	0.515
	1.03	[0.97-1.09]	0.316	1.04	[0.95-1.14]	0.366	1.05	[0.97-1.14]	0.208
	1.00	[0.95-1.07]	0.873	1.02	[0.94-1.11]	0.585	1.00	[0.91-1.09]	0.939
fever	1.04	[0.98-1.10]	0.227	1.01	[0.93-1.09]	0.884	1.11	[1.02-1.20]	0.017
	0.98	[0.93-1.04]	0.606	1.03	[0.95-1.13]	0.430	1.00	[0.92-1.08]	0.918
	1.03	[0.96-1.10]	0.376	1.02	[0.94-1.11]	0.610	1.04	[0.94-1.15]	0.463
brain_fog	1.06	[1.00-1.13]	0.057	1.09	[1.01-1.18]	0.032	1.04	[0.95-1.14]	0.405
	1.10	[1.04-1.17]	0.002	1.17	[1.07-1.28]	0.001	1.09	[1.00-1.19]	0.045
	1.05	[0.98-1.12]	0.136	1.09	[1.00-1.19]	0.061	1.01	[0.91-1.12]	0.860
eye_soreness	1.06	[1.00-1.13]	0.061	1.06	[0.97-1.15]	0.185	1.09	[0.99-1.19]	0.074
	1.05	[0.99-1.12]	0.114	1.08	[0.99-1.19]	0.102	1.07	[0.98-1.17]	0.108
	1.06	[0.99-1.14]	0.092	1.08	[0.98-1.19]	0.111	1.04	[0.94-1.16]	0.465
skipped_meals	1.15	[1.07-1.23]	<0.0001	1.14	[1.04-1.25]	0.006	1.18	[1.07-1.29]	0.001
	1.25	[1.17-1.33]	<0.0001	1.22	[1.11-1.35]	<0.0001	1.28	[1.17-1.40]	<0.0001
	1.17	[1.09-1.26]	<0.0001	1.24	[1.14-1.36]	<0.0001	1.08	[0.96-1.20]	0.195
unusual_muscle_pains	1.23	[1.15-1.31]	<0.0001	1.25	[1.15-1.36]	<0.0001	1.24	[1.13-1.38]	<0.0001
	1.31	[1.23-1.40]	<0.0001	1.33	[1.21-1.46]	<0.0001	1.36	[1.23-1.49]	<0.0001
	1.05	[0.98-1.13]	0.165	1.03	[0.94-1.14]	0.517	1.09	[0.97-1.22]	0.150
shortness_of_breath	1.20	[1.12-1.29]	<0.0001	1.29	[1.18-1.41]	<0.0001	1.10	[0.99-1.23]	0.065
	1.20	[1.12-1.28]	<0.0001	1.28	[1.16-1.41]	<0.0001	1.17	[1.06-1.29]	0.002
	1.00	[0.92-1.08]	0.909	0.94	[0.85-1.05]	0.277	1.07	[0.94-1.20]	0.302
feeling_down	1.04	[0.96-1.11]	0.335	1.01	[0.92-1.11]	0.810	1.06	[0.95-1.19]	0.277
	1.01	[0.94-1.09]	0.85	1.04	[0.93-1.16]	0.473	0.99	[0.89-1.10]	0.848
	1.10	[1.02-1.19]	0.02	1.10	[1.00-1.22]	0.058	1.11	[0.98-1.25]	0.090
ear_ringing	1.18	[1.10-1.26]	<0.0001	1.20	[1.09-1.32]	<0.0001	1.20	[1.08-1.33]	0.001
	1.18	[1.09-1.26]	<0.0001	1.16	[1.04-1.29]	0.005	1.26	[1.14-1.39]	<0.0001
	1.00	[0.93-1.08]	0.990	1.00	[0.90-1.11]	0.942	1.01	[0.90-1.14]	0.809
diarrhoea	1.04	[0.97-1.12]	0.294	1.04	[0.95-1.15]	0.409	1.03	[0.93-1.15]	0.519
	1.06	[0.98-1.14]	0.125	0.98	[0.88-1.09]	0.698	1.14	[1.03-1.25]	0.010
chest_pain	1.10	[1.02-1.18]	0.020	1.09	[0.99-1.20]	0.075	1.12	[0.99-1.26]	0.064
	1.17	[1.09-1.25]	<0.0001	1.21	[1.11-1.32]	<0.0001	1.15	[1.04-1.29]	0.009

	1.20	[1.12-1.29]	<0.0001	1.25	[1.13-1.38]	<0.0001	1.22	[1.10-1.35]	<0.0001
	1.06	[0.97-1.15]	0.200	1.04	[0.93-1.16]	0.489	1.09	[0.96-1.24]	0.185
abdominal_pain	1.14	[1.05-1.23]	0.001	1.12	[1.01-1.25]	0.032	1.18	[1.05-1.33]	0.005
	1.24	[1.14-1.34]	<0.0001	1.23	[1.09-1.38]	0.001	1.30	[1.16-1.45]	<0.0001
	1.07	[0.99-1.15]	0.112	1.10	[1.00-1.21]	0.061	1.02	[0.89-1.16]	0.771
swollen_glands	1.01	[0.94-1.09]	0.746	1.02	[0.93-1.13]	0.619	1.03	[0.91-1.16]	0.655
	1.03	[0.95-1.11]	0.501	1.06	[0.95-1.17]	0.293	1.04	[0.93-1.17]	0.450
	1.01	[0.93-1.10]	0.764	0.98	[0.87-1.09]	0.660	1.06	[0.93-1.21]	0.379
nausea	1.22	[1.13-1.32]	<0.0001	1.18	[1.06-1.31]	0.002	1.28	[1.14-1.44]	<0.0001
	1.23	[1.14-1.33]	<0.0001	1.18	[1.05-1.32]	0.005	1.31	[1.17-1.47]	<0.0001
	1.03	[0.95-1.12]	0.460	1.07	[0.97-1.19]	0.174	0.96	[0.85-1.10]	0.570
earache	1.00	[0.93-1.08]	0.953	1.01	[0.91-1.11]	0.894	1.02	[0.91-1.15]	0.725
	1.08	[1.00-1.17]	0.040	1.07	[0.96-1.19]	0.240	1.17	[1.04-1.30]	0.006
	1.00	[0.91-1.10]	0.972	0.95	[0.85-1.07]	0.404	1.07	[0.92-1.24]	0.365
delirium	1.13	[1.04-1.22]	0.005	1.09	[0.98-1.21]	0.132	1.18	[1.04-1.34]	0.012
	1.06	[0.98-1.16]	0.157	1.01	[0.90-1.15]	0.817	1.11	[0.98-1.26]	0.091
	1.10	[1.00-1.22]	0.062	1.10	[0.96-1.26]	0.179	1.13	[0.97-1.33]	0.124
skin_burning	1.07	[0.98-1.18]	0.150	1.10	[0.97-1.25]	0.155	1.07	[0.93-1.24]	0.342
	1.23	[1.12-1.36]	<0.0001	1.29	[1.13-1.48]	<0.0001	1.24	[1.09-1.42]	0.001
	1.07	[0.96-1.19]	0.235	1.04	[0.90-1.19]	0.581	1.13	[0.95-1.35]	0.170
irregular_heartbeat	1.09	[0.99-1.21]	0.090	1.09	[0.95-1.24]	0.216	1.11	[0.95-1.30]	0.193
	1.10	[0.99-1.22]	0.067	1.16	[1.01-1.34]	0.039	1.10	[0.94-1.28]	0.235
OR = Odds ratio; CI = Confidence interval									

Supplementary Table 9. Odds Ratio of experiencing each symptom in individuals with second dose and infected within 3 months compared to individuals with time since vaccination (TSV) between 3-4, 4-5, 5-6 months for the Omicron period.

	**All age groups**			**Younger adults (18-59 years)**			**Older adults (60+ years)**		
	**OR**	**95% CI**	**p-value**	**OR**	**95% CI**	**p-value**	**OR**	**95% CI**	**p-value**
runny_nose	1.34	[1.29-1.38]	<0.0001	1.22	[1.15-1.29]	<0.0001	1.40	[1.34-1.45]	<0.0001
	1.60	[1.54-1.66]	<0.0001	0.95	[0.85-1.06]	0.383	1.74	[1.66-1.81]	<0.0001

	1.49	[1.40-1.59]	<0.0001	1.04	[0.87-1.24]	0.703	1.60	[1.49-1.71]	<0.0001
	1.16	[1.13-1.20]	<0.0001	1.07	[1.02-1.13]	0.005	1.21	[1.17-1.25]	<0.0001
headache	1.30	[1.25-1.34]	<0.0001	1.03	[0.93-1.13]	0.571	1.38	[1.33-1.43]	<0.0001
	1.26	[1.19-1.33]	<0.0001	1.03	[0.88-1.20]	0.699	1.35	[1.27-1.43]	<0.0001
	1.33	[1.29-1.37]	<0.0001	1.18	[1.12-1.23]	<0.0001	1.42	[1.37-1.47]	<0.0001
fatigue	1.63	[1.58-1.69]	<0.0001	1.10	[1.00-1.21]	0.051	1.74	[1.68-1.80]	<0.0001
	1.70	[1.60-1.79]	<0.0001	1.16	[1.00-1.35]	0.054	1.83	[1.72-1.95]	<0.0001
	1.17	[1.13-1.20]	<0.0001	1.08	[1.03-1.13]	0.002	1.22	[1.17-1.26]	<0.0001
sneezing	1.26	[1.21-1.30]	<0.0001	0.88	[0.80-0.97]	0.009	1.34	[1.30-1.40]	<0.0001
	1.18	[1.12-1.25]	<0.0001	0.81	[0.70-0.94]	0.006	1.28	[1.21-1.36]	<0.0001
	1.30	[1.27-1.34]	<0.0001	1.21	[1.16-1.27]	<0.0001	1.35	[1.31-1.40]	<0.0001
persistent_cough	1.61	[1.56-1.66]	<0.0001	1.20	[1.10-1.31]	<0.0001	1.69	[1.63-1.75]	<0.0001
	1.60	[1.52-1.69]	<0.0001	1.26	[1.10-1.44]	0.001	1.68	[1.59-1.79]	<0.0001
	1.25	[1.21-1.28]	<0.0001	1.14	[1.09-1.20]	<0.0001	1.30	[1.25-1.35]	<0.0001
sore_throat	1.38	[1.33-1.42]	<0.0001	0.98	[0.89-1.08]	0.656	1.46	[1.41-1.52]	<0.0001
	1.31	[1.24-1.38]	<0.0001	0.99	[0.85-1.15]	0.864	1.39	[1.31-1.48]	<0.0001
	1.18	[1.13-1.23]	<0.0001	1.12	[1.05-1.20]	0.001	1.21	[1.15-1.28]	<0.0001
loss_of_smell	1.24	[1.18-1.30]	<0.0001	1.05	[0.93-1.18]	0.433	1.30	[1.24-1.38]	<0.0001
	1.26	[1.16-1.36]	<0.0001	1.16	[0.96-1.40]	0.116	1.31	[1.20-1.43]	<0.0001
	1.29	[1.25-1.33]	<0.0001	1.20	[1.14-1.25]	<0.0001	1.34	[1.30-1.40]	<0.0001
chills_or_shivers	1.46	[1.41-1.51]	<0.0001	1.14	[1.04-1.25]	0.004	1.54	[1.48-1.60]	<0.0001
	1.48	[1.40-1.57]	<0.0001	1.23	[1.07-1.42]	0.004	1.58	[1.48-1.68]	<0.0001
	1.24	[1.20-1.29]	<0.0001	1.16	[1.10-1.23]	<0.0001	1.29	[1.23-1.35]	<0.0001
altered_smell	1.37	[1.32-1.43]	<0.0001	1.12	[1.01-1.23]	0.031	1.47	[1.40-1.53]	<0.0001
	1.34	[1.25-1.43]	<0.0001	1.15	[0.98-1.34]	0.080	1.44	[1.34-1.55]	<0.0001
	1.12	[1.09-1.16]	<0.0001	1.06	[1.01-1.11]	0.022	1.16	[1.12-1.21]	<0.0001
dizzy_light_headed	1.30	[1.26-1.35]	<0.0001	0.99	[0.91-1.09]	0.909	1.38	[1.33-1.44]	<0.0001
	1.27	[1.20-1.35]	<0.0001	1.01	[0.88-1.17]	0.880	1.35	[1.27-1.45]	<0.0001
	1.23	[1.20-1.27]	<0.0001	1.17	[1.12-1.23]	<0.0001	1.27	[1.22-1.31]	<0.0001
hoarse_voice	1.38	[1.33-1.42]	<0.0001	1.03	[0.95-1.13]	0.484	1.45	[1.40-1.50]	<0.0001
	1.34	[1.27-1.41]	<0.0001	1.00	[0.88-1.15]	0.968	1.43	[1.35-1.52]	<0.0001

fever	1.28	[1.24-1.32]	<0.0001	1.24	[1.18-1.31]	<0.0001	1.30	[1.25-1.36]	<0.0001
	1.43	[1.38-1.49]	<0.0001	1.26	[1.15-1.39]	<0.0001	1.49	[1.43-1.55]	<0.0001
	1.51	[1.42-1.60]	<0.0001	1.34	[1.16-1.55]	<0.0001	1.59	[1.48-1.70]	<0.0001
brain_fog	1.16	[1.13-1.20]	<0.0001	1.08	[1.03-1.14]	0.001	1.23	[1.18-1.28]	<0.0001
	1.32	[1.27-1.38]	<0.0001	1.08	[0.99-1.19]	0.093	1.39	[1.34-1.46]	<0.0001
	1.30	[1.22-1.39]	<0.0001	0.97	[0.84-1.12]	0.681	1.42	[1.32-1.53]	<0.0001
eye_soreness	1.14	[1.10-1.18]	<0.0001	1.07	[1.01-1.13]	0.024	1.18	[1.13-1.23]	<0.0001
	1.21	[1.16-1.26]	<0.0001	1.02	[0.92-1.12]	0.767	1.26	[1.21-1.32]	<0.0001
	1.17	[1.10-1.25]	<0.0001	0.89	[0.76-1.03]	0.123	1.26	[1.18-1.36]	<0.0001
skipped_meals	1.18	[1.13-1.23]	<0.0001	1.17	[1.10-1.26]	<0.0001	1.18	[1.12-1.24]	<0.0001
	1.34	[1.28-1.41]	<0.0001	1.32	[1.17-1.49]	<0.0001	1.34	[1.27-1.41]	<0.0001
	1.47	[1.36-1.58]	<0.0001	1.35	[1.12-1.62]	0.001	1.49	[1.38-1.62]	<0.0001
unusual_muscle_pains	1.18	[1.14-1.22]	<0.0001	1.13	[1.07-1.19]	<0.0001	1.22	[1.16-1.28]	<0.0001
	1.35	[1.29-1.41]	<0.0001	1.18	[1.07-1.30]	0.001	1.42	[1.35-1.49]	<0.0001
	1.35	[1.25-1.44]	<0.0001	1.25	[1.08-1.46]	0.004	1.40	[1.30-1.52]	<0.0001
shortness_of_breath	1.17	[1.13-1.22]	<0.0001	1.13	[1.07-1.20]	<0.0001	1.21	[1.15-1.27]	<0.0001
	1.27	[1.21-1.33]	<0.0001	1.15	[1.03-1.28]	0.011	1.31	[1.25-1.38]	<0.0001
	1.25	[1.16-1.34]	<0.0001	0.99	[0.84-1.17]	0.904	1.34	[1.23-1.46]	<0.0001
feeling_down	1.05	[1.01-1.10]	0.018	1.00	[0.94-1.07]	0.988	1.09	[1.03-1.15]	0.002
	1.17	[1.11-1.23]	<0.0001	1.02	[0.90-1.15]	0.803	1.19	[1.12-1.25]	<0.0001
	1.16	[1.07-1.25]	<0.0001	0.91	[0.76-1.11]	0.354	1.21	[1.11-1.32]	<0.0001
ear_ringing	1.12	[1.08-1.17]	<0.0001	1.05	[0.99-1.11]	0.142	1.17	[1.11-1.22]	<0.0001
	1.20	[1.15-1.26]	<0.0001	1.03	[0.92-1.15]	0.652	1.29	[1.22-1.35]	<0.0001
	1.09	[1.01-1.18]	0.022	1.06	[0.90-1.26]	0.485	1.15	[1.06-1.25]	0.001
diarrhoea	1.14	[1.09-1.19]	<0.0001	1.13	[1.06-1.21]	<0.0001	1.14	[1.08-1.20]	<0.0001
	1.27	[1.21-1.34]	<0.0001	1.18	[1.04-1.34]	0.008	1.29	[1.23-1.36]	<0.0001
	1.34	[1.24-1.45]	<0.0001	1.21	[1.01-1.45]	0.039	1.40	[1.28-1.52]	<0.0001
chest_pain	1.07	[1.02-1.11]	0.001	1.08	[1.02-1.14]	0.011	1.05	[1.00-1.11]	0.050
	1.15	[1.10-1.20]	<0.0001	1.03	[0.92-1.15]	0.589	1.20	[1.14-1.26]	<0.0001
	1.21	[1.12-1.31]	<0.0001	1.02	[0.86-1.21]	0.827	1.28	[1.17-1.40]	<0.0001
abdominal_pain	1.10	[1.05-1.15]	<0.0001	1.07	[1.00-1.15]	0.056	1.12	[1.05-1.18]	<0.0001

	1.19	[1.13-1.25]	<0.0001	1.25	[1.10-1.42]	<0.0001	1.20	[1.13-1.27]	<0.0001
	1.18	[1.08-1.28]	<0.0001	1.07	[0.88-1.30]	0.495	1.23	[1.12-1.35]	<0.0001
swollen_glands	1.14	[1.10-1.18]	<0.0001	1.11	[1.05-1.17]	<0.0001	1.16	[1.11-1.22]	<0.0001
	1.21	[1.16-1.27]	<0.0001	1.05	[0.95-1.16]	0.344	1.29	[1.23-1.36]	<0.0001
	1.16	[1.07-1.24]	<0.0001	1.07	[0.91-1.25]	0.417	1.23	[1.13-1.34]	<0.0001
nausea	1.17	[1.11-1.22]	<0.0001	1.13	[1.05-1.21]	0.001	1.19	[1.12-1.27]	<0.0001
	1.34	[1.27-1.42]	<0.0001	1.32	[1.16-1.50]	<0.0001	1.36	[1.28-1.44]	<0.0001
	1.37	[1.25-1.50]	<0.0001	1.27	[1.05-1.54]	0.015	1.42	[1.28-1.57]	<0.0001
earache	1.13	[1.09-1.17]	<0.0001	1.08	[1.02-1.15]	0.014	1.16	[1.10-1.22]	<0.0001
	1.17	[1.12-1.23]	<0.0001	1.02	[0.92-1.14]	0.692	1.24	[1.18-1.31]	<0.0001
	1.16	[1.07-1.26]	<0.0001	1.11	[0.94-1.32]	0.204	1.21	[1.11-1.33]	<0.0001
delirium	1.14	[1.09-1.20]	<0.0001	1.06	[0.98-1.14]	0.126	1.23	[1.15-1.31]	<0.0001
	1.30	[1.23-1.38]	<0.0001	0.97	[0.85-1.11]	0.657	1.37	[1.29-1.47]	<0.0001
	1.24	[1.13-1.37]	<0.0001	0.85	[0.69-1.05]	0.130	1.33	[1.19-1.48]	<0.0001
skin_burning	1.15	[1.09-1.22]	<0.0001	1.08	[0.99-1.19]	0.087	1.19	[1.10-1.28]	<0.0001
	1.21	[1.13-1.30]	<0.0001	1.02	[0.86-1.20]	0.847	1.26	[1.17-1.36]	<0.0001
	1.29	[1.16-1.44]	<0.0001	1.10	[0.86-1.40]	0.463	1.38	[1.22-1.56]	<0.0001
irregular_heartbeat	1.07	[1.01-1.14]	0.014	1.08	[0.99-1.18]	0.080	1.06	[0.99-1.14]	0.110
	1.16	[1.08-1.24]	<0.0001	1.10	[0.94-1.29]	0.221	1.20	[1.11-1.29]	<0.0001
	1.09	[0.97-1.22]	0.145	1.01	[0.79-1.30]	0.912	1.15	[1.01-1.30]	0.037

OR = Odds ratio; CI = Confidence interval

Supplementary Figure 1. Odds ratio of asymptomatic infection, duration of symptoms ≥ 4 weeks, duration of symptoms ≥ 12 weeks, severe condition (two out of three among severe shortness of breath, fatigue, and fever), and hospitalization in app participants following booster vaccination vs 2nd dose, adjusted by age, BMI, sex, frailty, and IMD and stratified by variant and age.

a) Delta period	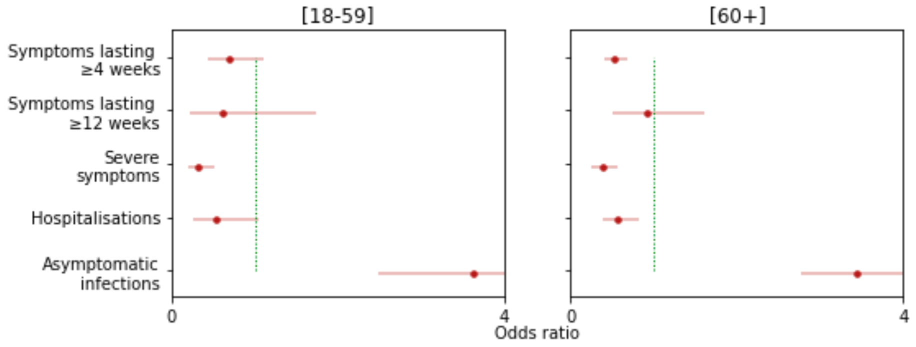
b) Omicron period	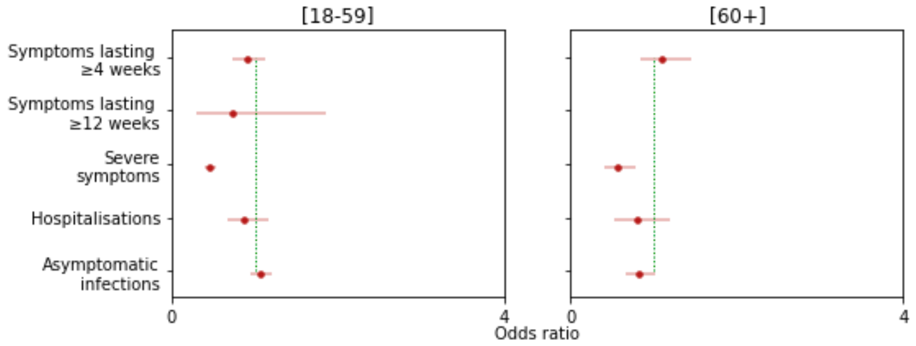

Supplementary Figure 2. Odds Ratio of individual symptoms in individuals vaccinated with third dose versus individuals vaccinated with the second dose adjusted by age, BMI, sex, frailty, and IMD for the (a) Delta period and (b) Omicron period stratified by age groups.

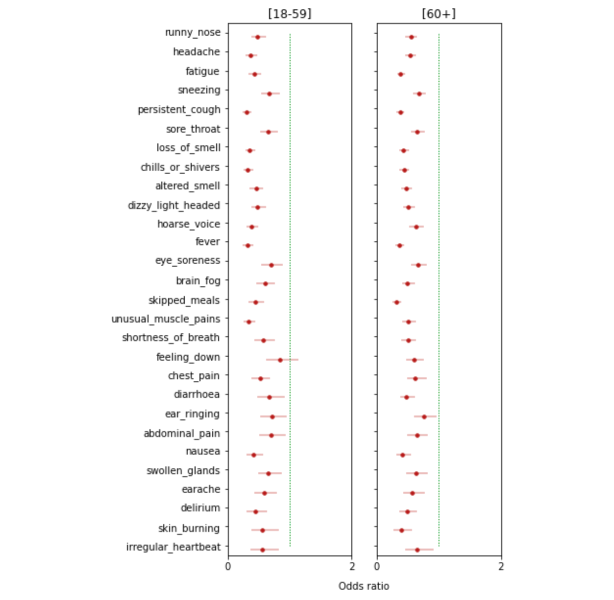

a) Delta period

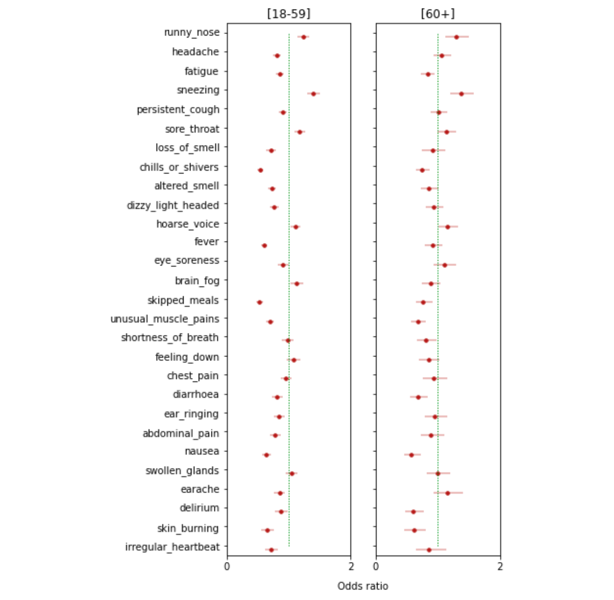

b) Omicron period

Supplementary Figure 3. Odds ratio of asymptomatic infection, duration of symptoms ≥ 4 weeks, severe condition (two out of three among severe shortness of breath, fatigue, and fever), and hospitalization in individuals vaccinated with second dose during the Delta period (a) and third dose during Omicron period (b) and infected within the first 3 months from vaccination versus individuals vaccinated with the second dose and infected within 3-4, 4-5, and 5-6 months from vaccination adjusted by age, BMI, sex, frailty, and IMD for the Delta period stratified by age.

a)

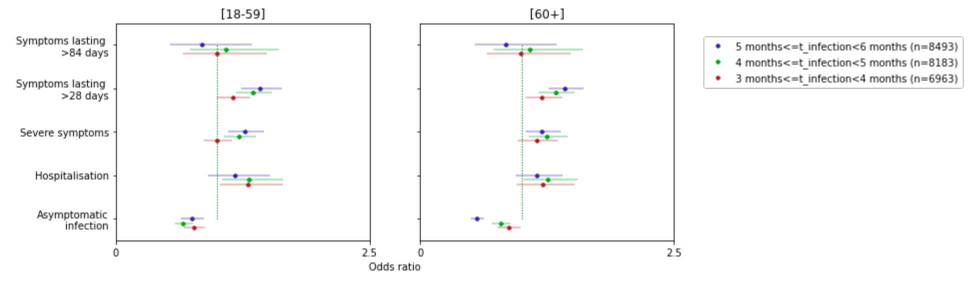

b)

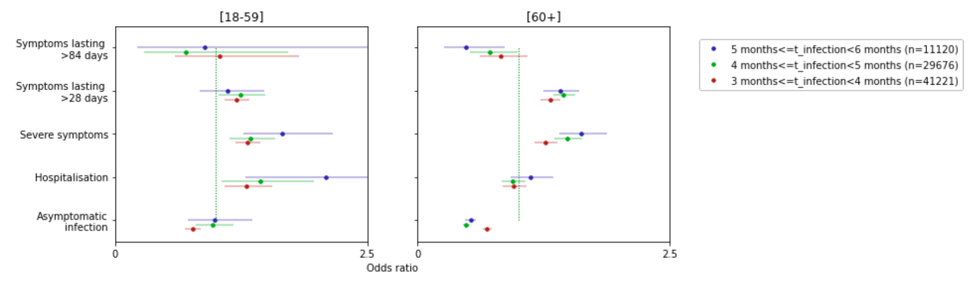

Supplementary Figure 4. Odds Ratio of individual symptoms in individuals vaccinated with second dose during Delta period (a) and third dose during Omicron period (b) and infected within the first 3 months from vaccination versus individuals vaccinated with the second dose and infected within 3-4, 4-5, and 5-6 months from vaccination adjusted by age, BMI, sex, frailty, and IMD, stratified by age group.

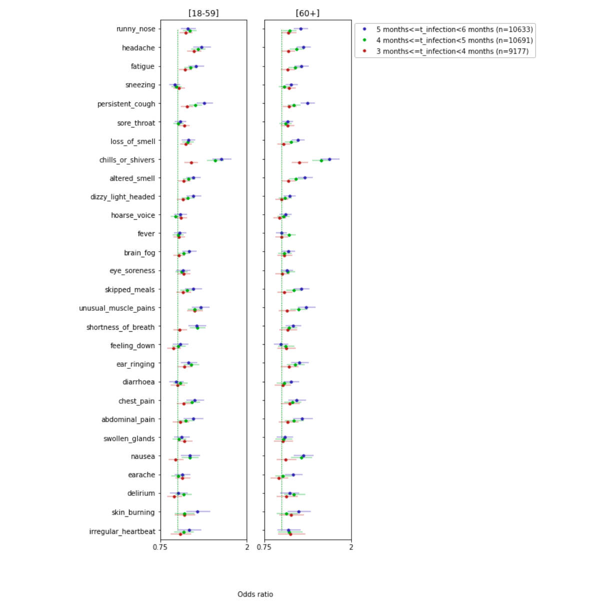

a) Delta period – Second dose

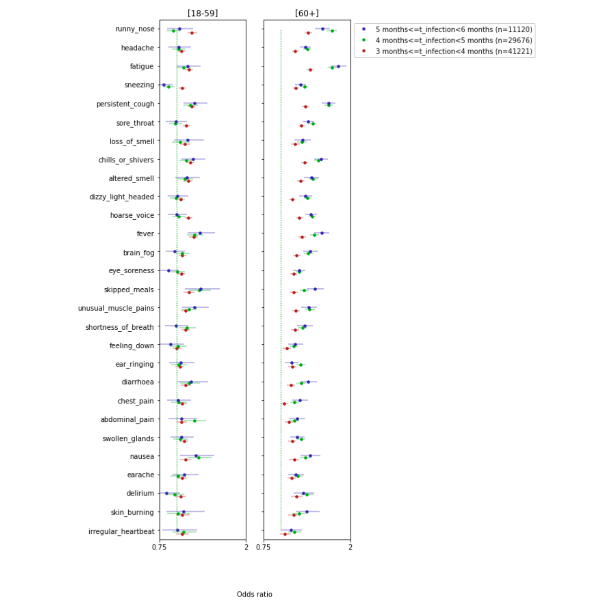

b) Omicron period – Third dose
